# Biomaterial-mediated macrophage polarization remodeling and sequential regulation: a potential strategy in bone infections treatment

**DOI:** 10.1038/s41413-025-00471-8

**Published:** 2025-11-25

**Authors:** Xiangwen Shi, Chao Xu, Zhian Chen, Mingjun Li, Zhe Yin, Bin Wang, Yang Li, Yipeng Wu, Xiaopei Wu, Yongqing Xu

**Affiliations:** 1 Kunming Medical University, Kunming, Yunnan China; 2 Department of Orthopedic Surgery, 920th Hospital of Joint Logistics Support Force of PLA, Kunming, Yunnan China; 3 Laboratory of Yunnan Traumatology and Orthopedics Clinical Medical Center, Kunming, Yunnan China; 4 College of Materials Science and Engineering, State Key Laboratory of New Textile Materials and Advanced Processing Technology, Wuhan Textile University, Wuhan, Hubei China; 5 State Key Laboratory of Advanced Technology for Materials Synthesis and Processing, Wuhan University of Technology, Wuhan, Hubei China

**Keywords:** Bone, Pulpitis

## Abstract

Osteomyelitis remains a global challenge in the field of orthopedics. Even after standard debridement and antibiotic-assisted treatment, the long-term recurrence rate remains at 20%-30%. Given the dynamic changes in immune responses and defense mechanisms during bone infection, as well as the complex “race for the surface” involving bacterial adhesion and host cells (macrophages and tissue cells) on implant surfaces, biomaterials with immunomodulatory functions have attracted considerable attention. Macrophages, as crucial components of the immune system, participate in the inflammatory regulation and tissue remodeling of bone infections through highly plastic polarization mechanisms after bacterial invasion. The different microenvironmental characteristics and therapeutic needs at different stages of bone infection highlight the promising applications of biomaterials capable of macrophage polarization remodeling and sequential regulation. In this review, we provide a detailed discussion of the complex immune regulatory patterns in the bone infection microenvironment and the critical functions of macrophage polarization. We then explore how implant surface properties influence bacterial adhesion and macrophage function, highlighting the importance of achieving precise and dynamic regulation of macrophage polarization based on the Race for the Surface theory. Furthermore, we focus on recent advances, potential challenges, and opportunities in biomaterial-mediated macrophage polarization remodeling and sequential modulation strategies across different stages of osteomyelitis, aiming to offer insights that may accelerate the clinical translation of novel biomaterial-based macrophage immunotherapies.

## Introduction

In the field of bone infections, osteomyelitis remains one of the most daunting challenges faced by orthopedic surgeons. With the increasing incidence of traumatic injuries from car accidents and the rising number of joint replacement surgeries, osteomyelitis has become a substantial medical burden.^1,2^ Bacteria are the primary pathogens of osteomyelitis, including *Staphylococcus aureus* (*S. aureus*), *Streptococcus pyogenes*, *Staphylococcus epidermidis* (*S. epidermidis*), and Enterococcus species, with *S. aureus* accounting for 75% of cases.^3^ Currently, even after thorough debridement surgery and full courses of antibiotic treatment, the recurrence rate of osteomyelitis remains at 20%-30%.^4^ The clinical treatment of osteomyelitis has become even more complex with the rising trend of infections caused by drug-resistant bacteria such as Pseudomonas aeruginosa and Klebsiella pneumoniae.

The successful colonization and proliferation of bacteria in bone tissue are closely linked to their various immune evasion mechanisms, including interference with and evasion from both innate and adaptive immune responses.^5,6^ Additionally, factors such as intracellular bacteria within the infection microenvironment, invasion of the osteocyte lacuno-canalicular network (OLCN), small colony variants (SCVs), persister cells, and biofilm formation further exacerbate antibiotic failure, making recurrent bone infections more likely.^7^ Macrophages play a crucial role in the innate immune response, as they can directly phagocytose pathogens or eliminate them through their highly adaptive phenotypic reprogramming ability.^8^ Macrophages can polarize into two main phenotypes, M1 and M2, contributing to enhanced pro-inflammatory and anti-inflammatory responses. M1 macrophages primarily secrete pro-inflammatory cytokines, facilitating immune activation and infection resistance, while M2 macrophages exhibit an anti-inflammatory phenotype, mainly participating in angiogenesis and bone tissue remodeling.^9^ Additionally, macrophage polarization directly or indirectly influences the activity of osteoblasts (OBs), osteoclasts (OCs), and mesenchymal stem cells (MSCs), playing a significant role in maintaining bone homeostasis and regulating infection responses.^10^ However, the balance between M1 and M2 macrophages is often disrupted due to bacterial invasion and biofilm formation in osteomyelitis.^11^

With the rapid advancement of advanced biomaterials, materials with direct antimicrobial properties have attracted widespread attention.^12^ They can enhance antimicrobial efficacy and reduce side effects on the human body by adjusting their composition, surface modification, and acting as carriers to deliver various antimicrobial agents such as antibiotics and antimicrobial peptides, through controlled release and targeted accumulation.^13,14^ Over the past few decades, modifying the physicochemical properties of biomaterial surfaces, such as topography, roughness, wettability, and surface charge, has been proven to be an effective antibacterial strategy for reducing bacterial adhesion and biofilm formation.^15^ However, due to the lack of an in-depth understanding of the dynamic changes in the immune microenvironment at different stages of bone infection, the implantation of biomaterials has shown suboptimal results in the long-term treatment of bone infections.^16^ Importantly, implants used for the treatment of osteomyelitis inevitably involve a “race for the surface” between bacteria and host cells such as tissue cells and macrophages on the material surface.^17^ It is currently widely accepted that the early occupation of the surface by tissue cells prior to bacterial adhesion, along with their rapid osseointegration with the implant, is key to winning this competition. Biomaterials may play a critical role in helping tissue cells gain the upper hand in this competition by modulating macrophage polarization toward the M1 or M2 phenotype, thereby contributing to both antibacterial defense and bone tissue regeneration. Over the past decade, inspired by the promising results of immunosuppressants in the treatment of mid- and late-stage tumors,^18^ biomaterials with immunomodulatory functions have shown potential in the treatment of bone infections. These materials may achieve both direct and immune-mediated antibacterial effects, thereby reducing the recurrence of infection. It is evident that biomaterials regulating the M1/M2 phenotype transition have greater potential than direct antimicrobial agents and are more in line with the practical requirements of the bone infection microenvironment. Paradoxically, while modifications to implant surface properties can regulate anti-inflammatory M2 polarization of macrophages and promote tissue integration, they may also facilitate bacterial adhesion and colonization. Overemphasizing the direct antibacterial properties of biomaterials, while neglecting their interactions with bacteria, tissue cells, and immune cells, may contribute to the persistence of infection. However, there is still a lack of comprehensive reviews on the research progress in this field. Therefore, a deep understanding of the entanglement between bacterial invasion and the host immune system, as well as the complex “race for the surface” among bacterial adhesion, tissue cell integration, and macrophage polarization on biomaterial surfaces, will facilitate the development of macrophage polarization-regulating biomaterials in the bone infection microenvironment.

**Fig. 1:**
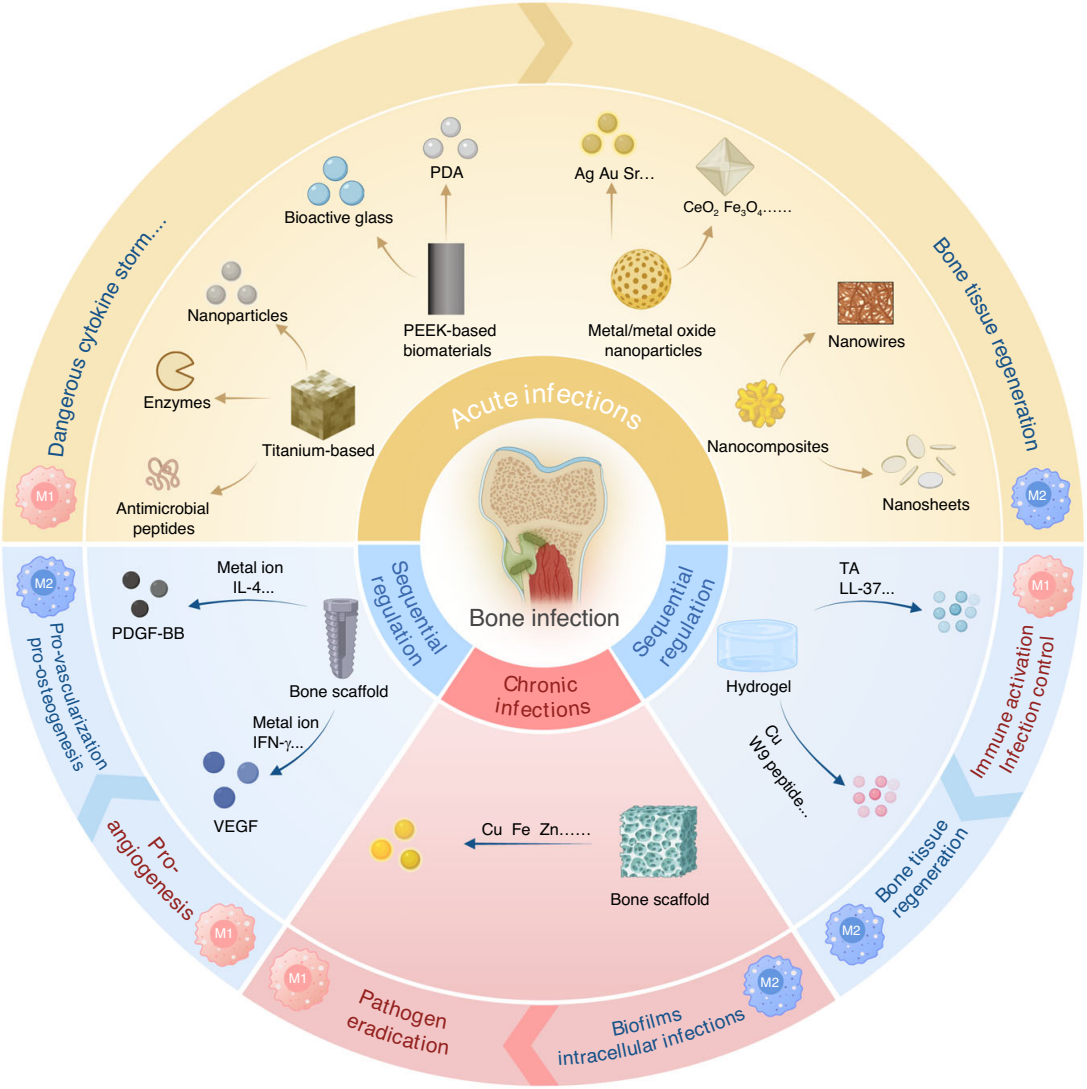
Biomaterial-mediated macrophage polarization remodeling and sequential regulation for the treatment of bone infections, including four main strategies: (1) In acute bone infections, biomaterials mediate M1 macrophage polarization to promote bone tissue repair and regeneration; (2) In chronic bone infections, biomaterials mediate M1 macrophages to facilitate infection eradication; (3) Sequential regulation of biomaterials from pro-inflammatory M1 to anti-inflammatory M2 macrophage polarization; (4) Sequential regulation by biomaterials from M1 pro-angiogenesis to M2 pro-vascularization and osteogenesis. Created with BioRender.com

In this review, we comprehensively discuss the research progress, challenges, and application prospects of biomaterials designed to regulate macrophage polarization, focusing on four main areas: (1) the immune evasion strategies employed by bacteria in the bone infection microenvironment and the role of macrophage polarization in maintaining bone homeostasis and combating infection; (2) the complex and multifaceted “race for the surface” among bacteria, tissue cells, and macrophages on the surface of biomaterials used for bone infection treatment; (3) recent advances in biomaterial-mediated macrophage polarization remodeling and sequential modulation strategies applied across different stages of bone infection; (4) the challenges and future prospects in the development of macrophage polarization-regulating biomaterials (Fig. 1).

## Immune regulation patterns during bone infection

**Fig. 2:**
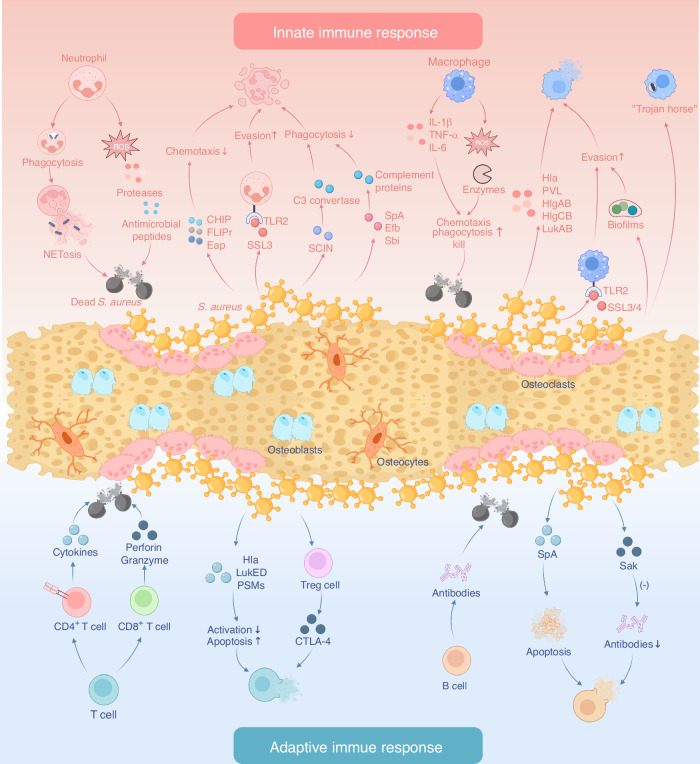
Immune defense processes in the bone infection microenvironment following pathogen invasion, and bacterial interference and evasion strategies. The upper half: Bactericidal mechanisms of neutrophils and macrophages after innate immune activation, and bacterial resistance strategies. The lower half: Bactericidal mechanisms of T cells and B cells during adaptive immune responses, and bacterial resistance strategies. Created with BioRender.com

The immune system plays a crucial role in maintaining bone homeostasis and defending against bone infections, involving both the innate and adaptive immune systems. Upon invasion of bone tissue by *S. aureus*, the immune system initiates a series of defense mechanisms, releasing cytokines and inflammatory factors. This section focuses on the defensive processes of immune system following pathogen invasion, as well as bacterial interference and evasion strategies (Fig. 2).

Innate immune cells, primarily macrophages and neutrophils, play an initial defensive role against pathogens through phagocytosis, secretion of pro-inflammatory factors, oxidative stress capture, and the formation of neutrophil extracellular traps (NETs) to kill planktonic bacteria.^5,19^ Generally, neutrophils, as highly active phagocytes, are rapidly recruited to the site of bone infection driven by chemotactic signals produced by host cells, where they then phagocytose and kill invading pathogens. They exert their immune functions mainly through four mechanisms: (1) production of reactive oxygen species (ROS), which cause oxidative damage to bacteria, directly inducing their death; (2) phagosomes produce various types of antimicrobial peptides with bacteriostatic or bactericidal activity; (3) neutrophils contain multiple proteases, such as cathepsin G, gelatinase, and collagenase, which degrade the protein components of *S. aureus*; (4) formation of NETs that release antimicrobial peptides and proteases, thereby inducing and killing bacteria.^20^

However, *S. aureus* interferes with neutrophil chemotaxis, activation, phagocytosis, and NET formation through a series of mechanisms, thereby inhibiting their bactericidal capabilities. During the recruitment stage of neutrophils in early infection, *S. aureus* secretes three virulence factors: chemotaxis inhibitory protein (CHIP), formyl peptide receptor-like 1 inhibitory protein (FLIPr), and extracellular adherence protein (Eap), which affect neutrophil chemotaxis. CHIP specifically impairs the formyl peptide response to reduce the production of chemotactic factors, thereby weakening neutrophil chemotaxis.^21^ FLIPr inhibits neutrophil chemotaxis by reducing intracellular calcium mobilization.^22^ Eap blocks neutrophil chemotaxis and recruitment by binding to intercellular adhesion molecule 1 (ICAM-1).^23^ These different virulence mechanisms make it difficult for neutrophils to rapidly chemotax and recruit to the infection site, significantly reducing the bactericidal effect of innate immunity. Subsequently, neutrophil activation relies on the recognition of *S. aureus* surface receptors. Staphylococcal superantigen-like 3 (SSL3) is another virulence factor secreted by *S. aureus*. It interferes with neutrophil recognition of pathogens by competitively binding to Toll-like receptor 2 (TLR2), thereby promoting bacterial immune evasion.^24,25^ Meanwhile, the Staphylococcal complement inhibitor secreted by *S. aureus* binds to C3 convertase, greatly reducing neutrophil phagocytosis and killing of *S. aureus*.^26^ In addition to impairing chemotaxis, FLIPr also mediates escape of neutrophil phagocytic.^22^ Additionally, *S. aureus* interferes with complement-mediated phagocytosis by capturing complement proteins through *S. aureus* protein A (SpA), extracellular fibrinogen-binding protein, and Staphylococcal IgG-binding molecule (Sbi).^27–29^ ROS are crucial for the formation and structure of NETs, but *S. aureus* can neutralize ROS and inhibit NET formation through staphyloxanthin and regulatory enzyme activity.^30^ Thus, *S. aureus* employs multiple immune strategies to evade capture and killing by neutrophils.

Macrophages are specialized phagocytes and an important source of cytokines. In bone infections, like neutrophils, macrophages are recruited from the bloodstream to the site of infection by chemotactic factors to kill invading pathogens. Specifically, under pathogen stimulation, TLRs initiate signal cascades upon recognizing pathogen-associated molecular patterns (PAMPs), producing various cytokines including IL-1β, tumor necrosis factor-α (TNF-α), and interferon-α. These inflammatory factors activate and recruit phagocytes, initiating the immune response.^31^ Additionally, macrophages destroy and phagocytize pathogens by releasing ROS, reactive nitrogen species (RNS), and enzymes, as well as mediating autophagy.^32–34^ For macrophage recruitment and phagocytosis, *S. aureus* employs various mechanisms to evade the immune response. It has been reported that SSL3 and SSL4 interfere with macrophage recognition of *S. aureus* by blocking TLR2 activation, thereby promoting bacterial immune evasion.^35^ Moreover, the formation of *S. aureus* biofilms creates a robust mechanical barrier that enhances immune evasion by shielding surface PAMPs, increasing resistance to macrophage invasion and phagocytosis.^36^ *S. aureus* can persist and exist within macrophages, termed “Trojan horse” macrophages, which have been shown to contribute to bacterial dissemination and progression.^37^ Additionally, various secreted factors, including pore-forming toxins such as α-hemolysin (Hla), Panton-Valentine leukocidin, γ-hemolysin (HlgAB, HlgCB), and leukocidin AB (LukAB), directly kill macrophages and neutrophils.^38,39^

During bone infections, adaptive immunity is activated, involving both T cell and B cell responses. Upon antigen activation, T cells differentiate into helper T cells (CD4 T cells) and cytotoxic T cells (CD8 T cells). CD4 T cells enhance the immune response by secreting cytokines that activate other immune cells and can also regulate the immune response to avoid excessive inflammation.^40^ CD8 T cells directly kill pathogens by secreting perforin and granzyme. As chronic osteomyelitis progresses, CD8 T cell function gradually declines, leading to the formation of exhausted CD8 T cells. Due to prolonged exposure to antigens and inflammatory factors, these cells lose their efficacy, reducing the production of cytokines such as IFN-γ and TNF-α, as well as the secretion of perforin and granzyme.^41^ *S. aureus* inhibits T cell function through multiple mechanisms, including interference with T cell activation and induction of apoptosis via pore-forming toxins such as Hla, LukED, and phenol-soluble modulins (PSMs), as well as immune suppression through upregulation of Treg cells.^42,43^ Additionally, *S. aureus* superantigens can induce massive T cell activation, subsequently triggering Treg cells to secrete CTLA-4, which interferes with normal immune responses.^44^ B cells combat *S. aureus* by producing antibodies, but *S. aureus* employs various immune evasion mechanisms. For instance, it induces B cell apoptosis via the secreted virulence factor SpA and degrades antibody IgG through staphylokinase (Sak), thereby inhibiting antibody-mediated immune responses.^45–47^ These mechanisms prevent the maintenance of antibody levels even after acute infection, impairing long-term immune memory and defense capabilities.

In conclusion, a thorough understanding of the immunoregulatory patterns and functions within the bone infection microenvironment, along with an improved comprehension of immune cell bactericidal mechanisms and pathogen immune evasion strategies, will facilitate the precise design and development of immunoregulatory antimicrobial biomaterials.

## Macrophage regulation in bone infections

### Biological functions of macrophage polarization

Macrophages, the first phagocytes to be discovered, play a crucial role in host defense mechanisms and immune cell functions primarily through the phagocytosis and killing of pathogens, clearing dead cells, and repairing damaged tissue. Due to high plasticity, they can undergo reprogramming in response to various environmental stimuli, such as inflammatory or infectious signals, thereby exhibiting distinct functional phenotypes.^48^

Similar to the nomenclature of Th1/Th2, early experimental studies categorized macrophages into two main polarization states: classically activated (M1) and alternatively activated (M2).^49^ Classically activated M1 macrophages are primarily induced by interferon-γ (IFN-γ), microbial products such as lipopolysaccharide (LPS), and TNF-α.^50^ Their characteristic biological features include the secretion of high levels of pro-inflammatory cytokines (TNF-α, IL-1β, and IL-6), nitric oxide (NO), and ROS, as well as the expression of major histocompatibility complex class II (MHC-II) molecules. These factors collectively enhance the bactericidal activity, antigen-presenting capability, and complement-mediated phagocytosis of macrophages.^51^ The activation and differentiation of M1 macrophages are further regulated by growth factors such as macrophage colony-stimulating factor (M-CSF) and granulocyte-macrophage colony-stimulating factor (GM-CSF).^52^

In contrast to the functions of M1 macrophages, M2 macrophages are induced by IL-4 and IL-13 and primarily promote the resolution of inflammation and tissue repair by suppressing inflammatory responses, activating anti-inflammatory Th2 cells, and clearing apoptotic cells. These macrophages selectively express the mannose receptor CD206, CD163, arginase 1 (Arg1), resistin-like molecules (such as Fizz1), IL-10, and TGF-β, and are capable of producing collagen precursors to facilitate tissue repair.^53,54^

With the advancement of in vitro experiments on human and mouse macrophages under various anti-inflammatory stimuli, M2 macrophages have been further subdivided into four subtypes (M2a, M2b, M2c, and M2d), each with specific biological functions and regulatory mechanisms. Specifically, M2a macrophages are induced and activated by IL-4 and IL-13, express high levels of IL-4R, CD163, and CD206, and produce inflammatory chemokines (CCL17, CCL18, CCL22, and CCL24) that are involved in tissue repair and remodeling. M2b macrophages are generated in response to immune complexes and TLR (Toll-like receptor) ligands, express high levels of TNF-α, IL-1β, IL-6, and IL-10, and participate in immune regulation, while secreting CCL1 to recruit regulatory T cells (Tregs).^55,56^ M2c macrophages, induced by IL-10, glucocorticoids, or TGF-β, express high levels of the scavenger receptor CD163 to clear dead cells and secrete large amounts of IL-10 and TGF-β to suppress immune responses, facilitating inflammation resolution and tissue repair.^57^ M2d macrophages, also known as tumor-associated macrophages (TAMs), are activated by TLR ligands and adenosine signaling, and have been shown to promote angiogenesis by expressing high levels of IL-10, VEGF, and low levels of IL-12.^58,59^ However, these M2 macrophage subtypes are not entirely distinct and may overlap in function and expression patterns. For a comprehensive understanding of macrophage activation in M1 and M2 paradigms, readers are advised to refer to a recent review article. Table 1 details the phenotypic characteristics of M1 and M2 macrophages as described in previous studies.

**Table 1:** Phenotypic characteristics of M1 and M2 macrophages

Phenotypic characteristics	M1 Macrophages	M2 Macrophages			
		M2a	M2b	M2c	M2d
Activation stimuli markers	IFN-γ, LPS, TNF-α	IL-4, IL-13, IL-10	Immune complexes+TLR ligands, IL-1β	IL-10, TGF-β, Glucocorticoids	IL-6, Adenosine, TLR agonists
Human	CD80, CD86, MHC-II, iNOS	CD206, CD163	CD86, MHC-II	CD163, CD206, MerTK	CD206, VEGF receptor
Mouse	CD80, CD86, MHC-II, iNOS	CD163, Arg-1, Ym1/2, Fizz1	CD86, MHC-II	CD163, CD206, MerTK, Arg-1	CD206, VEGF receptor
Cytokine profile					
Human	TNF-α, IL-1β, IL-6, IL-12	IL-10, TGF-β, IL-1ra	IL-10 high, IL-1β, TNF-α	IL-10 high, TGF-β	IL-10, VEGF, IL-12 low, TNF-α low
Mouse	TNF-α, IL-1β, IL-6, IL-12	IL-10, TGF-β, IL-1ra	IL-10 high, IL-1β, TNF-α	IL-10 high, TGF-β	IL-10, VEGF, IL-12 low, TNF-α low
Chemokines					
Human	CCL5, CXCL5, CXCL9, CXCL10	CCL17, CCL18, CCL22, CCL26	CCL1, CCL20	CCL6, CCL18, CXCL13	CCL5, CCL10, CXCL16
Mouse	CCL5, CXCL5, CXCL9, CXCL10	CCL17, CCL18, CCL22, CCL23, CCL26	CCL1, CCL20	CCL6, CCL18, CXCL13	CCL5, CCL10, CXCL16
Function	Microbicidal, pro-inflammatory responses, tissue damage	Tissue repair, extracellular matrix deposition, anti-parasitic responses	Immune regulation, moderate inflammation, pathogen clearance	Immunosuppression, resolution of inflammation, tissue remodeling	Angiogenesis, tumor progression, tissue repair
Key transcription factors	IRF5, STAT1	STAT6, PPAR-γ			
Reactive species production	High levels of ROS and RNS	Low ROS, high antioxidant activity			
Role in bone infection	Pathogen clearance, exacerbation of inflammation	Resolution of inflammation, tissue regeneration			

*Arg-1* Arginase-1, *CCL* Chemokine (C-C motif) ligand, *CXCL* Chemokine (C-X-C motif) ligand, *Fizz1* Found in inflammatory zone 1, *IFN-γ* Interferon-gamma, *IL* Interleukin, *iNOS* Inducible nitric oxide synthase, *IRF5* Interferon regulatory factor 5, *LPS* Lipopolysaccharide, *MHC-II* Major histocompatibility complex class II, *PPAR-γ*: Peroxisome proliferator-activated receptor gamma, *RNS* Reactive nitrogen specie, *ROS* Reactive oxygen species, *STAT* Signal transducer and activator of transcription, *TGF-β* Transforming growth factor beta, *TLR* Toll-like receptor, *TNF-α* Tumor necrosis factor alpha, *VEGF* Vascular endothelial growth factor

For decades, the M1/M2 dichotomy and the terms “classical activation” and “alternative activation” have been widely used by researchers. It is noteworthy that the M1 and M2 phenotypes are not opposing but coexisting states, with their mixed phenotypes often determined by stimuli from the tissue microenvironment. Current characterizations of M1/M2 are mainly based on in vitro studies and may not necessarily correlate with the mixed phenotypes of macrophages in vivo.^60^ Hence, the binary model of pro-inflammatory M1 macrophages and anti-inflammatory M2 macrophages appears to be overly simplistic, and there is still no consensus on how to standardize the definition of macrophage polarization in both in vitro and in vivo disease models. In response, immunologists summarized macrophage polarization nomenclature and experimental guidelines at the International Congress of Immunology in 2013.^61^ In this review, we describe M1/M2 polarization as the general activation patterns of macrophages.

### Macrophage polarization and regulation of bone homeostasis

**Fig. 3:**
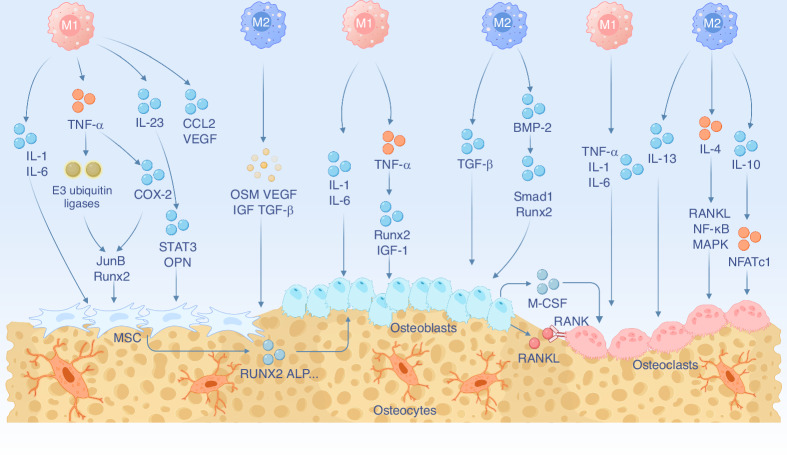
Schematic illustration of the role of macrophage polarization in regulating bone homeostasis, including the regulation of MSCs, osteoblasts, and osteoclasts by different macrophage polarization phenotypes (M1 and M2). Created with BioRender.com

Bone homeostasis refers to the balanced state between bone formation and resorption activities, ensuring bone repair and remodeling.^62^ Macrophages play a crucial role in bone homeostasis, and their polarization state directly or indirectly affects the activities of OBs, OCs, and MSCs during bone remodeling (Fig. 3). M1 macrophages primarily promote the bone resorptive activity of OCs through pro-inflammatory responses, whereas M2 macrophages promote bone formation and tissue repair through anti-inflammatory effects and the secretion of osteogenic factors.

#### M1/M2 macrophages and OBs

OBs are the key cells responsible for bone formation, derived from MSCs, and are primarily involved in the synthesis, secretion, and mineralization of bone tissue. Macrophages are distributed in the bone marrow, on the bone surface, and around mature bone cells, playing a crucial role in maintaining bone homeostasis by directly or indirectly regulating the function of OBs.^63^

During their maturation process, M1 macrophages primarily secrete pro-inflammatory factors TNF-α and IL-6 to regulate OB activity. In bone tissue, TNF-α inhibits osteoblast differentiation and bone formation by reducing alkaline phosphatase (ALP) activity and suppressing IGF-1 expression.^64^ Additionally, TNF-α inhibits Runx2 stability by modulating the expression of Smurf1 and Smurf2, thereby hindering osteogenesis.^65^ Interestingly, low levels of TNF-α can promote osteogenic differentiation, showing a dose-dependent effect and indicating its dual role in osteoblast function.^66^ IL-6, a key member of the interleukin family, regulates osteogenic differentiation by binding to the IL-6 receptor. Mechanistically, IL-6 inhibits osteoblast differentiation in vitro in a dose-dependent manner through the SHP2/MEK2/ERK and SHP2/PI3K/Akt2 pathways.^67^ Consistent with the dual effects of TNF-α, it has been reported that exogenous IL-6 and soluble IL-6 receptor (IL-6sR) synergistically enhance the osteogenic effects of BMP-7 on rat OBs.^68^

M2 macrophages significantly promote the differentiation and activity of OBs by secreting osteoinductive factors such as BMP-2, BMP-4, and TGF-β. The anti-inflammatory environment created by M2 macrophages enhances the proliferation, adhesion, and mineralization capacities of OBs, while upregulating the expression of osteogenic-related genes such as Runx2, ALP, osteocalcin (OCN), and osteopontin (OPN).^69^ For instance, BMP-2 binds to its receptor and activates the SMAD1 signaling pathway, promoting the nuclear translocation of Runx2. This induces the expression of ALP, OPN and OCN, enhancing osteogenic differentiation and mineralization.^70^ Thus, macrophages regulate the functional activity of OBs by releasing pro-inflammatory factors and osteoinductive factors, thereby maintaining normal bone homeostasis.

#### M1/M2 macrophages and OCs

OCs are the key cells responsible for bone resorption and originate from the same progenitor cells as macrophages, with differentiation competition between them playing a regulatory role in bone resorption. M1 macrophages secrete pro-inflammatory cytokines, such as TNF-α and IL-1β, which promote osteoclast formation. TNF-α is the most potent osteoclastogenic factor produced by M1 macrophages; it not only directly promotes the differentiation of osteoclast precursors but also synergistically enhances RANKL-induced osteoclastogenesis by activating the NF-κB and PI3K/Akt signaling pathways.^71^ IL-1 also plays a critical role in bone resorption by inducing the release of TNF-α and IL-6, further stimulating osteoclast activity.^72^ As an important member of the IL-1 family, IL-1β has been shown to promote osteoclast formation by increasing RANKL production and directly stimulating the differentiation of osteoclast precursors.^73^ Moreover, upon binding to its receptor, IL-6 activates the JAK/STAT3 signaling pathway, inducing osteoclastogenesis and regulating bone resorption through the NFATc1 transcription factor.^74,75^ However, the precise regulatory mechanisms by which TNF-α, IL-1β, and IL-6 secreted by M1 macrophages influence osteoclast functional activity remain incompletely understood, necessitating further investigation using co-culture systems.

M2 macrophages inhibit osteoclast formation by secreting anti-inflammatory cytokines such as IL-4, IL-10, and IL-13. IL-4 and IL-13 downregulate RANKL expression and upregulate OPG via a STAT6-dependent signaling pathway, thereby reducing osteoclast activity.^76^ In addition to suppressing RANKL expression, IL-4 directly prevent osteoclast formation by inhibiting the activation of NF-κB and MAPK signaling.^77^ Furthermore, IL-10 reduces bone resorption by inhibiting osteoclastogenesis through interference with NFATc1 activation and nuclear translocation.^78^

#### M1/M2 macrophages and MSCs

MSCs are multipotent stem cells capable of differentiating into OBs, adipocytes and chondrocytes, playing a critical role in bone tissue repair and regeneration. Changes in macrophage polarization significantly impact the proliferation, differentiation, and functional regulation of MSCs. M1 macrophages exert complex regulatory effects on MSCs. Zhao et al.^79^ reported that TNF-α induces the expression of E3 ubiquitin ligases, promoting the degradation of key osteogenic transcription factors such as Runx2 and JunB, thereby inhibiting MSC osteogenic differentiation. Although pro-inflammatory cytokines secreted by M1 macrophages may suppress MSC osteogenesis, early inflammation can transiently enhance MSC osteogenic potential via M1-associated pro-inflammatory stimulation. Ekström et al.^80^ found that TLR activation/LPS stimulation of human monocytes enhances the osteogenic differentiation of MSCs. Further mechanistic studies revealed that M1 macrophages promote new bone formation in specific environments, likely mediated by their secretion of prostaglandin E2 (PGE2) and COX-2 signaling, potentially activating the OSM/gp130 pathway to promote MSC osteogenesis.^81,82^ Another study indicated that IL-23 secreted by M1 macrophages induces MSC osteogenesis through activation of the STAT3/β-catenin pathway.^83^ Additionally, cytokines CCL2 and VEGF facilitate bone tissue repair by promoting MSC recruitment.^84^ Similar to OBs, M1 macrophages also exhibit a dual regulatory effect on the osteogenic differentiation of MSC, and further investigation into the underlying mechanisms is still required.

M2 macrophages play a critical role in bone regeneration, promoting MSC migration, proliferation, and osteogenic differentiation through their anti-inflammatory properties and secretion of factors such as TGF-β1, IL-4, and IL-10. A previous study have shown that co-culturing M2 macrophages with MSCs significantly enhances MSC osteogenic differentiation in vitro, accompanied by upregulation of growth factors TGF-β1, VEGF, and IGF-1.^85^ Zhang et al.^86^ observed that IL-4-loaded hydrogel scaffolds activate the TGF-β1/Smad pathway, inducing MSCs to upregulate osteogenesis-related genes, such as Runx2, OCN, and ALP, and promote the formation of mineralized bone nodules. Additionally, M2 macrophages enhance BMSC osteogenic differentiation through exosome-mediated upregulation of IL-10, a process facilitated by activating the IL-10/IL-10R signaling pathway.^87^ Interestingly, under inflammatory conditions, MSCs secrete factors like PGE2, which promote the polarization of M2 macrophages and enhance their anti-inflammatory functions.^88,89^ The reciprocal regulation between M2 macrophages and MSCs contributes to a microenvironment conducive to bone tissue repair, promoting bone regeneration.

In summary, the dynamic balance between M1 and M2 macrophage polarization is crucial for maintaining normal bone homeostasis. Whether M1 macrophages inhibit osteogenesis or promote bone repair may depend on the specific quantity and type of cytokines they secrete. Under conditions of low-level or early-stage inflammation, M1 macrophages may enhance MSC osteogenic differentiation, an aspect that should not be overlooked in the design of osteogenic biomaterials. Moreover, the complex immune mechanisms involved in bone homeostasis are challenging to quantify under a unified standard. Future research should explore therapeutic strategies and biological mechanisms for optimizing bone remodeling and repair by regulating macrophage polarization.

### Regulation of macrophage polarization in bone infections

Macrophages exhibit high plasticity and dynamic adaptability, allowing them to switch between pro-inflammatory M1 and anti-inflammatory M2 subtypes in the infection environment to counteract pathogen invasion and regulate tissue repair. During bone infection, the distribution and polarization of macrophage subtypes are significantly affected. Previous studies have examined the in vivo distribution of macrophage subtypes using immunohistochemical analysis of cryosections from biopsy samples of patients with chronic osteomyelitis and normal bone marrow. In approximately 50% of biopsies from chronic osteomyelitis patients, a reduction or absence of macrophage subtypes was observed.^90^ The distribution of macrophage subtypes in post-traumatic osteomyelitis is affected and primarily localized to the site of infection.^91^ In a comparative study of patients with post-traumatic osteomyelitis before and after treatment, Peters et al.^92^ found that inflammatory macrophages were absent or present in minimal numbers before treatment, while anti-inflammatory macrophages increased after treatment. These findings suggest that anti-inflammatory macrophages may play a crucial role in the downregulation of inflammation, whereas the absence of inflammatory macrophages may impact the early immune response in patients with traumatic osteomyelitis. Overall, the local suppression or abnormal distribution of macrophage subtypes may be a key factor contributing to the persistence of inflammation during bone infection, while the specific mechanisms remain unclear.

Given the dynamic changes and complexity of macrophage subtypes in vivo, immunologists have made significant progress in in vitro studies related to macrophage polarization in bone infections. In a three-dimensional co-culture model simulating biomaterial-associated infection (BAI), Giraldo-Osorno et al.^93^ found that M1 macrophages inhibit osteogenesis by downregulating osteogenesis-related genes in osteocytes, such as OCN and phosphate-regulating neutral endopeptidase on chromosome X (PHEX), upregulating anti-mineralization genes like matrix extracellular phosphoglycoprotein (MEPE), and increasing the expression of the pro-osteoclastogenic factor RANKL. Interestingly, M1 macrophages also exhibit a compensatory pro-osteogenic effect by downregulating the bone inhibitory factor sclerostin (SOST) and upregulating the expression of BMP-2. M2 macrophages primarily upregulate the anti-osteoclastogenic gene OPG, demonstrating anti-resorptive effects. These findings suggest that communication between macrophage polarization and osteocytes may mediate the dysregulation of osteoblast-osteoclast coupling under BAI conditions.

Mechanistically, macrophage polarization and function are regulated by multiple signaling pathways. Activation of STAT1 promotes M1 polarization and enhances pro-inflammatory functions, while activation of STAT3 and STAT6 promotes M2 polarization and enhances tissue repair through IL-4, IL-13, and IL-10 signaling.^94^ STAT6 enhances M2 macrophage function by inhibiting NF-κB/HIF-1α-dependent transcription.^95^ Zhu et al.^96^ found that SETD2 expression is downregulated in macrophages of patients with acute pyogenic osteomyelitis, and the loss of SETD2 promotes M1 polarization and increases glycolytic activity. Further mechanistic studies revealed that overexpression of SETD2 catalyzes H3K36me3 and binds to the HIF-1α gene, thereby suppressing HIF-1α expression, which reverses the M1 polarization and enhanced glycolysis effects caused by SETD2 deficiency. Additionally, STAT-mediated macrophage activation is regulated by SOCS family members.^97^ In a model of septic arthritis caused by *S. aureus* infection, inhibition of TLR2, the binding site of *S. aureus*, reduced joint inflammatory damage and bacterial load by regulating the STAT1/STAT3/SOCS3 pathway, accompanied by downregulation of NF-κB activity.^98^ In a recent study on infection-associated fracture fixation (IAFF), Dai et al.^99^ demonstrated that miR-345-3p promotes the transition of M1 to M2 macrophages and reduces the expression of pro-inflammatory cytokines by inhibiting MAPK kinase kinase 1 (MAP3K1) and the NF-κB pathway. These findings suggest that targeting the regulation of STAT, NF-κB, and MAPK signaling pathways to influence macrophage phenotypes may represent an important therapeutic target for bone infections.

In chronic osteomyelitis, bacterial biofilms disrupt the M1/M2 balance by altering macrophage metabolism and polarization. Before biofilm formation, host immune cells primarily rely on glycolysis for energy, a metabolic pathway that supports the polarization of macrophages towards the pro-inflammatory M1 phenotype, contributing to early anti-infective responses.^57^ However, as biofilms form and bacterial load significantly increases, glucose and other nutrients in the microenvironment become increasingly scarce. To adapt to this nutrient-deficient state, macrophage metabolism gradually shifts towards oxidative phosphorylation to meet energy demands. Metabolic changes and the barrier function of *S. aureus* biofilms promote the transition of macrophages to the M2 phenotype, accompanied by an upregulation of anti-inflammatory factors and a reduction in antimicrobial peptides.^11,100^ This process accelerates early fibrotic responses, leading to abscess formation. In conclusion, regulating the imbalance of macrophage polarization and metabolism, particularly through interventions targeting biofilms, may represent a promising strategy for treating chronic bone infections.

## Interactions between host tissue cells and bacteria on biomaterial surfaces

After the implantation of the implant, a competition arises between microbial colonization and tissue integration on the implant surface, a dynamic referred to as the “race for the surface” by orthopedic surgeon Gristina.^101^ Over time, this competition between host tissue cells and bacteria has gradually become a significant research focus in the field of bone infections. When bacteria infect the implant, they adhere to its surface through various adhesion mechanisms, often leading to the formation of biofilms. In this process, if tissue cells, aided by the host immune system, can achieve effective bone integration on the biomaterial surface, the implant may be protected from bacterial infection. However, the results are often less than ideal. As previously mentioned, although the immune system can mount an effective response, bacteria rapidly gain the upper hand on the implant surface through various interference and immune evasion mechanisms, leading to persistent clinical infections and implant failure, even threatening patient lives.^102^ Therefore, developing biomaterials and implants associated with infections presents significant challenges. Furthermore, the formation of biofilms not only protects bacteria from antimicrobial agents but also allows them to evade the surveillance of the host immune system, making the eradication of infections more difficult.^103^ Therefore, studying and exploring the interactions between host cells (such as tissue cells and macrophages) and bacteria on biomaterial surfaces helps us understand the underlying mechanisms of bone infections and provides a theoretical foundation for the development of immunomodulatory biomaterials.

### “Love and hate” relationship between material properties and macrophage fate

In immunotherapy for treating bone bacterial infections, macrophages, as key regulators of immune responses, are profoundly influenced by the properties of biomaterials.^104^ The initial interaction between implanted biomaterials and macrophages is often determined by the surface characteristics of the material, such as morphology, roughness, wettability, charge, and chirality. These physical parameters not only directly affect macrophage adhesion, migration, and polarization, but also indirectly guide macrophage functions by modulating the interactions between the material surface and the cell membrane.^105^ For instance, surface morphology can influence cell shape and cytoskeleton reorganization, while wettability can alter protein adsorption patterns, thereby affecting the direction of immune responses. Hence, understanding how the properties of materials regulate macrophage fate is crucial for designing biomaterials with immunomodulatory functions. In the following sections, we will explore the roles of physical characteristics (surface morphology and roughness), surface chemistry (wettability, charge, and chirality), material composition, material degradation, and physical stimulation in macrophage regulation (Table 2).

**Table 2:** Influence of material physical properties on macrophage functions

Physical properties	Material property	Macrophage polarization	Regulatory mechanism	Ref.
Surface topography	SLA topographies	Promoting M1 polarization	SLA topographies can modulate expression of proinflammatory cytokines and chemokines, including TNF-α, IL-1β, IL6, MCP-1, and MIP-1α	^107^
	SLA topographies	Promoting M2 polarization	SLA topographies can upregulate the macrophage attractant chemokines MIP-1a and MCP-1, while reduce the secretion of the M1-typical chemokine IP-10	^108^
	Honeycomb-like TiO₂	Promoting M2 polarization	Honeycomb-like TiO₂ can enhance M2 macrophage polarization through the activation of the RhoA/Rho-associated protein kinase signaling pathway, promoting the expression of anti-inflammatory cytokines such as IL-4 and IL-10, ultimately facilitating new bone formation and osseointegration	^109^
	Submicron forest-like silicon surface	Promoting M2 polarization	Submicron forest-like silicon surface can increase the expression of M2 polarization markers (CD163 and CD206) compared to smooth surfaces, aiding in vitro osteogenic differentiation and in vivo bone formation	^110^
	2D surface topography	Promoting M2 polarization	Macrophages can exhibit maximal adhesion and elongation on grooves with a width of 500 nm, with cytoskeletal reorganization along the groove direction, showing stronger M2 polarization	^113^
	Nanofibers	Reducing M1 polarization	PU-nano causes minimal macrophage responses (M1 polarization) in vitro and in vivo and induced only mild foreign body reactions compared to PU-micro membranes.	^114^
	3D electrospun PCL nanofiber scaffolds	Promoting M2 polarization	3D Scaffolds can promote deeper macrophage migration, increases the M2/M1 macrophage ratio in a 4-week rat model, and induce angiogenesis and tissue regeneration	^115^
	3D-printed chitosan scaffolds	Promoting M1 polarization	3D-printed chitosan scaffolds with large pores can significantly induce higher levels of pro-inflammatory cytokines, such as TNF-α, IL-12, and IL-23, from human monocytes/macrophages	^116^
	3D-printed bioceramic scaffolds	Promoting M2 polarization	3D-printed bioceramic scaffolds with an ordered arrangement can significantly enhance M2 polarization of macrophages both in vivo and in vitro, subsequently promoting BMSC migration and osteogenic differentiation	^117^
	3D substrates	Reducing M1 polarization	Co-culture of MSCs with THP-1-derived macrophages on 3D substrates can significantly reduce the production of soluble factors related to inflammation and chemotaxis, including IL-6 and MCP-1	^118^
Surface wettability	Hydrophilic surface	Promoting M2 polarization	Hydrophilic surface can enhance albumin adsorption, thereby driving M2 macrophage polarization and releasing anti-inflammatory cytokines	^119^
	Coating hydrophilic material on titanium dioxide surface	Promoting M2 polarization	Hydrophilic surface can promote the polarization of RAW 264.7 cells toward the M2 phenotype by enhancing fibronectin adsorption and deposition, activating the PI3K and NF-κB signaling pathways	^120^
	Hydrophilic rough titanium	Promoting M2 polarization	Hydrophilic rough titanium can induce macrophage activation similar to the anti-inflammatory M2-like state, increasing levels of interleukins IL-4 and IL-10	^121^
	Hydrophilic carbon nanofibers	Reducing M1 polarization	Hydrophilic carbon nanofibers can significantly reduce pro-inflammatory cytokine secretion	^122^
	Superhydrophilic titanium nanotubes	Promoting M2 polarization	Superhydrophilic titanium nanotubes can induce RAW 264.7 cells to secrete anti-inflammatory factors such as BMP-2, IL-10, and TGF-β, while reducing LPS-induced pro-inflammatory cytokine secretion	^126^
Surface charge	Strontium-containing hydroxyapatite (Sr-HAp) into hydroxypropyl chitosan/aldehyde dextran hydrogels	Promoting M2 polarization	Hydrogels can promote M2 macrophage polarization and bone regeneration through the controlled release of Sr^2+^	^130^
	Modification of divalent cations (Ca and Sr) on titanium surface	Transition from M1 to M2 phenotype	Titanium can induce the transition of macrophages from the M1 to M2 phenotype, reducing early inflammatory responses while producing cytokines that promote the osteogenic differentiation of MSCs	^131^
	Lithium-doped nano-hydroxyapatite hydrogels	Promoting M2 polarization	Hydrogels can continuously release lithium ions, which induce M2 macrophage polarization by activating the JAK1/STAT6/STAT3 signaling pathway, further promoting bone repair	^132^
	Cationic gelatin scaffolds	Promoting M1 polarization	Cationic gelatin scaffolds can stimulate RAW 264.7 cells to secrete IL-12 via the TLR-4 signaling pathway, enhancing Th1 responses in vivo	^134^
	a-NFC	Promoting M1 polarization	a-NFC can promote higher levels of TNF-α	^136^
	CNCs	Promoting M1 polarization	CNCs can promote M1 polarization by enhancing the expression of pro-inflammatory cytokines and chemokines.	^137^
	Cellulose nanoparticle-modified 3D-printed chitosan/silk fibroin composite scaffolds	Transition from M1 to M2 phenotype	Composite scaffolds can promote the transition of M1 to M2 macrophages, enhancing both in vivo and in vitro osteogenic differentiation	^138^
	Nano-hydroxyapatite particles	Promoting M2 polarization	Nano-hydroxyapatite particles can promote the transformation of human macrophages to the M2 phenotype and facilitate the secretion of the anti-inflammatory cytokine IL-10 by activating the transcription factor c-Maf	^139^
Surface chirality	Enantiomers of NIBC	Promoting M1 polarization	The number of macrophages adhering to the L-NIBC surface is significantly higher than that on the D-NIBC surface, and these cells exhibit M1-like morphological changes	^144^
	CoFe₂O₄/poly (vinylidene fluoride-trifluoroethylene) films	Promoting M2 polarization	CoFe₂O₄/poly films can significantly downregulate M1 polarization responses in BMDMs while promoting the expression of M2 polarization markers	^145^

*a-NFC* Carboxymethylated-nanofibrillated cellulose films, *BMP-2* Bone morphogenetic protein-2, *CNCs* Anionic cellulose nanocrystals, *IL* Interleukin, *IP-10* Interferon gamma-induced protein 10, *JAK1* Janus kinase 1, *MCP-1* Monocyte chemoattractant protein-1, *MIP-1α* Macrophage inflammatory protein-1 alpha, *MSCs* Mesenchymal stem cells, *NIBC* N-isobutyryl-L(D)-cysteine, *PCL* Polycaprolactone, *PU* Polyurethane, *RhoA* Ras homolog family member A, *ROCK* Rho-associated protein kinase, *SLA* Sandblasting and acid etching, *HAp* Hydroxyapatite, *STAT* Signal transducer and activator of transcription, *TGF-β* Transforming growth factor beta, *TLR-4* Toll-like receptor 4, *TNF-α* Tumor necrosis factor alpha

#### Surface topography and roughness

The surface topography of biomaterials is a critical factor in determining macrophage behavior, with micro- and nanoscale surface features affecting macrophage adhesion, migration, and polarization.^106^ Different surface topographies can regulate macrophage behavior by influencing the formation of the cytoskeleton and adhesion points. Disordered or rough surfaces may more readily induce an M1 pro-inflammatory response, which is crucial for early infection control. Refai et al.^107^ found that, as surface roughness increased on polished and mechanically treated titanium, macrophage adhesion and spreading significantly improved, along with enhanced secretion of pro-inflammatory cytokines such as IL-1β, IL-6, and TNF-α. Their findings suggested that rough surface topographies tend to activate the M1 macrophage phenotype, promoting an early inflammatory response. However, the effect of roughness is not linear; moderate roughness can promote bone healing by modulating macrophage polarization. Barth et al.^108^ demonstrated that sandblasted and acid-etched titanium surfaces could induce RAW 264.7 macrophages to polarize toward the M2 phenotype, thereby promoting wound healing and bone regeneration. Zhu et al.^109^ reported that a small-scale honeycomb-like TiO_2_ surface (90 nm) enhanced M2 macrophage polarization through the activation of the RhoA/Rho-associated protein kinase signaling pathway, promoting the expression of anti-inflammatory cytokines such as IL-4 and IL-10, ultimately facilitating new bone formation and osseointegration. Recently, Sun et al.^110^ introduced a submicron forest-like silicon surface, which significantly increased the expression of M2 polarization markers (CD163 and CD206) compared to smooth surfaces, aiding in vitro osteogenic differentiation and in vivo bone formation. Moreover, macrophage polarization is jointly regulated by surface hydrophilicity and roughness. Increasing both roughness and hydrophilicity on titanium surfaces can promote M2 polarization, enhancing bone formation and tissue repair through the regulation of the Wnt signaling pathway.^111^

Furthermore, the two-dimensional (2D) and three-dimensional (3D) surface topographies of materials provide distinct microenvironments for macrophages, significantly influencing their functions.^112^ On 2D surfaces, microstructural topography directly impacts macrophage behavior. Chen et al.^113^ found that different groove widths on polycaprolactone (PCL), polylactic acid (PLA), and polydimethylsiloxane (PDMS) surfaces had significant effects on the morphology and polarization of RAW 264.7 macrophages. Specifically, on grooves with a width of 500 nm, macrophages exhibited maximal adhesion and elongation, with cytoskeletal reorganization along the groove direction, showing stronger M2 polarization. As the groove width increased to 2 μm, macrophage adhesion and fusion decreased, indicating that 2D surface topography can regulate macrophage behavior through microstructural changes. At smaller scales, nanofiber materials exhibited lower inflammatory responses and milder foreign body reactions. Compared to microfiber surfaces, nanofibers, due to their smaller diameters, reduced M1 macrophage pro-inflammatory polarization, leading to fewer foreign body reactions during long-term implantation.^114^ Compared to 2D surfaces, 3D structures better mimic the complex tissue environment in vivo, thereby more effectively modulating macrophage behavior. In an in vivo study, expanded 3D electrospun PCL nanofiber scaffolds, due to their higher porosity, promoted deeper macrophage migration, increased the M2/M1 macrophage ratio in a 4-week rat model, and induced angiogenesis and tissue regeneration.^115^ These results suggested that 3D geometries, by controlling pore size and layer thickness, can provide deeper interactions for macrophages, enhancing their functionality. In contrast, 2D electrospun nanofiber membranes only exhibited surface-adherent macrophages. Furthermore, Almeida et al.^116^ found that 3D-printed chitosan scaffolds with large pores significantly induced higher levels of pro-inflammatory cytokines, such as TNF-α, IL-12, and IL-23, from human monocytes/macrophages, indicating that 3D structures not only affect macrophage migration but also significantly regulate their polarization and immune response. A recent study highlighted that 3D-printed bioceramic scaffolds with an ordered arrangement significantly enhanced M2 polarization of macrophages both in vivo and in vitro, compared to randomly arranged structures, subsequently promoting BMSC migration and osteogenic differentiation.^117^ Moreover, compared to 2D monolayer cultures, co-culture of MSCs with THP-1-derived macrophages on 3D substrates significantly reduced the production of soluble factors related to inflammation and chemotaxis, including IL-6 and MCP-1, highlighting the critical role of the topographical cues in modulating intercellular communication between macrophages and MSCs.^118^

In summary, material surface topography influences macrophage fate through multiple mechanisms: 2D surfaces primarily regulate macrophage polarization by altering adhesion and morphology, while 3D surfaces provide more complex microenvironments, promoting deeper cell migration and functional expression. Additionally, nanoscale features further enhance this regulatory effect. Therefore, by precisely designing surface topography and roughness, particularly by integrating 2D and 3D structures, the immune response of macrophages can be effectively controlled, optimizing the application of biomaterials in treating bone bacterial infections.

#### Surface wettability

The surface wettability of materials significantly influences the fate of macrophages, potentially affecting their immune response by modulating monocyte/macrophage adhesion. Visalakshan et al.^119^ found that hydrophilic material surfaces enhance albumin adsorption, thereby driving M2 macrophage polarization and releasing anti-inflammatory cytokines. Lv et al.^120^ further demonstrated that coating hydrophilic material on titanium dioxide surface promotes the polarization of RAW 264.7 cells toward the M2 phenotype by enhancing fibronectin adsorption and deposition, activating the PI3K and NF-κB signaling pathways. In contrast, hydrophobic materials tend to inhibit protein adsorption, inducing an M1 pro-inflammatory response.^121,122^ Chun et al.^123^ found that hydrophilic carbon nanofibers significantly reduced pro-inflammatory cytokine secretion, while hydrophobic materials enhanced T-cell activation. Therefore, rational control of material surface wettability can achieve controllable regulation of macrophage polarization.

Moreover, the surface wettability of materials can be dynamically regulated by external stimuli (e.g., electrical, thermal, or light induction), allowing reversible switching between hydrophilicity and hydrophobicity.^124,125^ This reversible wettability regulation provides a means to adjust the M1/M2 macrophage response according to practical needs. Hydrogenation of titanium nanotubes can make their surface superhydrophilic, inducing RAW 264.7 cells to secrete anti-inflammatory factors such as BMP-2, IL-10, and TGF-β, while reducing LPS-induced pro-inflammatory cytokine secretion.^126^ Overall, modulating material surface wettability can effectively influence macrophage polarization, guiding immune responses toward pathogen clearance and tissue repair. Combined with other surface characteristic regulation strategies (such as roughness and morphology), this approach is expected to further optimize the functionality of materials in clinical applications.

#### Surface charge

It has been reported that the cell membranes of macrophages generally carry a negative charge, with a potential range of approximately −10 to −90 mV.^127^ The surface charge of biomaterials not only significantly affects macrophage adhesion, proliferation, and protein adsorption and release but also regulates the bone immune environment.^128^ Generally, positively charged (cationic) materials are more effective than negatively charged (anionic) materials in inducing an anti-inflammatory response in macrophages and promoting M2 polarization.^129^ Ding et al.^130^ incorporated strontium-containing hydroxyapatite (Sr-HAp) into hydroxypropyl chitosan/aldehyde dextran hydrogels, which not only improved mechanical properties but also promoted M2 macrophage polarization and bone regeneration through the controlled release of Sr^2+^. Another study showed that chemical modification of divalent cations (Ca and Sr) on titanium surfaces induced the transition of macrophages from the M1 to the M2 phenotype, reducing early inflammatory responses while producing cytokines that promote the osteogenic differentiation of MSCs.^131^ Luo et al.^132^ reported that a lithium-doped nano-hydroxyapatite hydrogel, which continuously released lithium ions, induced M2 macrophage polarization by activating the JAK1/STAT6/STAT3 signaling pathway, further promoting bone repair. Interestingly, positively charged biomaterials are more easily phagocytosed and internalized by macrophages compared to neutral or negatively charged materials. Miller et al.^133^ studied the endocytosis of liposomes with different surface charges (including neutral, positively charged, and negatively charged) in J774 macrophages and found that positively charged liposomes were more easily engulfed by macrophages than negatively charged ones. However, cations may also promote anti-tumor effects by inducing M1 polarization. Polylysine, polyethyleneimine (PEI), and cationic gelatin scaffolds can stimulate RAW 264.7 cells to secrete IL-12 via the TLR-4 signaling pathway, enhancing Th1 responses in vivo.^134^

Negatively charged materials typically induce M1 macrophage polarization. The functional group modulation of surface charge on nanocellulose films has a significant impact on monocyte/macrophage polarization, such as grafting with carboxymethyl (anionic) or hydroxypropyltrimethyl ammonium (cationic) groups.^135^ A previous study have shown that carboxymethyl cellulose (CMC) films can induce M1 polarization of monocytes/macrophages, while cellulose films modified with hydroxypropyltrimethyl ammonium do not induce M1 polarization, possibly due to the inertness of the material.^136^ Samulin et al.^137^ reported that anionic cellulose nanocrystals (CNCs) could promote M1 polarization by enhancing the expression of pro-inflammatory cytokines and chemokines. Furthermore, the surface charge of nanoparticles (NPs) has been shown to significantly affect macrophage polarization. Cellulose nanoparticle-modified 3D-printed chitosan/silk fibroin (SF) composite scaffolds promoted the transition of M1 to M2 macrophages, enhancing both in vivo and in vitro osteogenic differentiation.^138^ Mahon et al.^139^ reported that nano-hydroxyapatite particles promoted the transformation of human macrophages to the M2 phenotype and facilitated the secretion of the anti-inflammatory cytokine IL-10 by activating the transcription factor cMaf. In summary, surface charge not only influences the interaction between biomaterials and macrophages but also significantly affects immune regulation through processes such as polarization and inflammatory response modulation.^140,141^ By controlling the surface charge of biomaterials, effective strategies can be developed to regulate macrophage immune responses, accelerating bone tissue regeneration or modulating immune antibacterial responses.

#### Surface chirality

Biological systems are inherently chiral, consisting of various chiral molecules such as D-glucose, L-amino acids, and helical DNA, which play crucial roles in maintaining the biological functions of cells and organisms.^142,143^ Introducing chiral properties to the surface of biomaterials can induce different macrophage phenotypes and immune responses. Sun et al.^144^ systematically investigated the adhesion behavior and activation states of human macrophages and neutrophils on smooth nano-structured silicon and polymer substrates using enantiomers of N-isobutyryl-L(D)-cysteine (L(D)-NIBC) as surface modifiers. The results demonstrated significant behavioral differences in macrophages on L-NIBC and D-NIBC surfaces. Specifically, the number of macrophages adhering to the L-NIBC surface was significantly higher than that on the D-NIBC surface, and these cells exhibited morphological changes indicative of a pro-inflammatory M1 phenotype. In contrast, macrophages on the D-NIBC surface maintained a round morphology, indicating an anti-inflammatory M2 phenotype. These findings suggest that surface chirality can serve as an important factor in regulating macrophage behavior, providing new insights for the design of immunomodulatory biomaterials. In further studies in this field, Kehr et al.^145^ explored the effect of surface chirality on the adhesion behavior of primary human macrophages in vitro by performing chiral selective functionalization of periodic mesoporous organosilica (PMO) materials modified with D(L)-mannose (D(L)-MAN). The results showed that the number of macrophages adhering to the PMO-D-MAN monolayer was approximately four times that on the PMO-L-MAN monolayer, indicating that macrophages can recognize not only surface functional groups but also surface chirality. This discrimination ability may be more pronounced in environments with biological macromolecules such as proteins and nucleic acids. Zhou et al.^146^ reported that the chirality-patterned potential distribution of CoFe₂O₄/poly (vinylidene fluoride-trifluoroethylene) films can significantly downregulate M1 polarization responses in BMDMs while promoting the expression of M2 polarization markers.

Recent studies have also explored the regulatory roles of chiral materials in specific immune pathways. Danelius and co-workers^147^ developed a novel A3 macrocyclization synthesis strategy to precisely control R- and S-configurations, studying their binding capabilities to the CD36 receptor and their effects on macrophage inflammatory responses through the TLR 2/6 pathway. Experiments in RAW 264.7 macrophages showed that dynamic chirality influenced inflammation-related outcomes such as NO production and the release of cytokines and chemokines. Furthermore, surface chirality can trigger protein adsorption and wettability changes on smart polymer surfaces, potentially further regulating immune responses.^148,149^ In conclusion, surface chirality significantly influences macrophage behavior and modulates different polarization states, highlighting its potential in the design of immunoregulatory materials.

#### Material composition

The composition of biomaterials plays a crucial role in the immunoregulation of macrophages. Biomaterials, such as bone cements, silicate/phosphate-based bioceramics, and ionically crosslinked hydrogels, influence macrophage behavior and function through their degradation products, including bioactive ions, proteins, and small bioactive molecules.^150,151^ Some ions that induce bone regeneration have been proven to participate in the regulation of macrophage polarization. Li et al.^152^ incorporated calcium silicate into calcium phosphate bone cement and demonstrated in vitro and in vivo that the composite bone cement promotes M2 macrophage polarization through the release of silicon, while also enhancing bone repair and rapid vascularization. Another study found that magnesium-organic framework-doped bone cement significantly induced the M2 phenotype of macrophages, promoting the osteogenic differentiation of BMSCs and the formation of calcium nodules.^153^ Xu et al.^154^ synthesized a multifunctional hydrogel through ionic crosslinking and hydrogen bonding interactions, which activated M2 macrophages, simultaneously promoting rapid anti-inflammatory responses and angiogenesis.

The composition of different biomaterials can also influence macrophage recognition and phagocytic behavior through surface modification strategies. Surface modification strategies for synthetic polymers, such as polystyrene microparticles, can significantly affect macrophage phagocytosis. Qi et al.^155^ found that polystyrene nanoparticles modified with polyethylene glycol or CD47 reduced the phagocytic activity of M1 macrophages. Additionally, negatively charged coatings, such as bovine serum albumin, exhibited lower phagocytic efficiency in in vitro experiments, while positively charged poly-L-lysine coatings enhanced macrophage phagocytosis of the particles.^156^ These studies indicate that the compositional properties of materials, such as surface charge and chemical modifications, can significantly alter the recognition and internalization processes of macrophages, thereby influencing their fate. Another material modification strategy involves the use of biologically derived cell membranes, such as macrophage membrane-coated nanoparticles. This strategy can effectively prolong the circulation time of particles in vivo, reduce recognition by phagocytes, and specifically target infection and inflammation sites.^157–159^ Moreover, combining other cell membranes, such as red blood cell membranes and leukocyte membranes, to form hybrid cell membrane nanoparticles can achieve more complex multifunctional modifications, further enhancing targeting capabilities.^160,161^ Therefore, future research should focus on elucidating the precise molecular mechanisms by which different material compositions regulate macrophage fate, and validate these mechanisms through systematic experiments to advance the development and clinical translation of novel immunoregulatory biomaterials.

#### Material degradation

The degradation characteristics of implanted materials have a profound impact on the immune response and fate of macrophages. The degradation process of materials typically occurs through physical and chemical interactions or through cell-mediated dissolution, hydrolysis, and enzymatic degradation.^162,163^ This process can alter the surface morphology, chemical composition, and mechanical properties of materials, providing persistent physical and chemical stimuli to macrophages, thereby regulating their polarization states. Huang et al.^164^ fabricated MXene-modified 3D-printed ceramic scaffolds, which promoted M1 polarization of macrophages in the early stages post-implantation, followed by a transition to an M2-dominated anti-inflammatory response, thus creating an immune microenvironment conducive to bone repair. Another study found that dexamethasone released from 3D-printed scaffolds significantly enhanced the phenotype switch of macrophages from M1 to M2, potentially promoting the osteogenic differentiation of co-cultured MSCs through soluble factors like BMP-2 and IL-6.^165^ However, the impact and mechanisms of biomaterial degradation properties on macrophage-MSC crosstalk remain a topic for further investigation and resolution.

In contrast, non-degradable or slowly degradable materials (such as crosslinked polymers or thermoplastic polymers) often lead to chronic inflammatory responses and fibrosis.^166^ For instance, uncoated polypropylene mesh materials can induce M1 macrophage responses in animal models, accompanied by significant macrophage aggregation on fiber surfaces. Conversely, polypropylene materials modified with extracellular matrix (ECM) hydrogel coatings can reduce M1 responses by releasing bioactive ECM fragments during degradation.^167,168^ Moreover, chemically crosslinked or non-degradable ECM scaffold materials can also trigger chronic inflammation and M1-type macrophage responses, whereas rapidly degradable ECM scaffolds can promote tissue reconstruction by inducing M2 responses.^169^ By studying the degradation processes of different material compositions and their effects on immune cells, we can gain a better understanding of how to leverage material design to regulate macrophage immune responses.

#### Physical stimulation

Numerous studies have revealed the impact of physical stimuli, such as electric fields, magnetic fields, and ultrasound, on the fate of macrophages.^170,171^ These stimuli exhibit great potential in tissue repair and inflammation regulation by modulating cellular behaviors, such as migration, phagocytic capacity, and polarization state.

Electrical stimulation (ES), as a controllable method, can induce macrophage polarization by regulating parameters such as voltage and frequency, particularly during the transition from the inflammatory to the remodeling phase of wound healing.^172^ Notably, a local electric field is generated when the epithelial barrier of injured human tissue is disrupted, which may regulate macrophage function.^173^ Mccann et al.^174^ successfully induced macrophages to polarize towards the M2 phenotype using a direct current electric field (12.7–30.5 V/s) and observed periodic increases in calcium ion concentration, closely associated with the formation of the M2 phenotype. Furthermore, Xu et al.^175^ observed an increase in the M2/M1 macrophage ratio under an electric field of 53 mV/mm using a non-contact ES device, further validating the regulatory effect of the electric field on macrophage polarization. Hoare et al.^176^ found that an electric field as low as 5 mV/mm could guide macrophages to migrate toward the anode, with the effect being strongest at 300 mV/mm. The electric field also significantly enhanced the phagocytic capacity of macrophages, including the uptake of apoptotic cells and pathogens, accompanied by the activation of the PI3K and ERK pathways, calcium mobilization, and cytokine secretion. This indicates that ES plays a crucial role in coordinating macrophage functions, clearing pathogens, and promoting healing. Another study utilized RNA-Seq to analyze the potential molecular and pathway mechanisms after treating RAW 264.7 macrophages with a direct current electric field (200 mV/mm). The results showed that the steroid biosynthesis pathway was most significantly affected by the electric field. The electric field enhanced the atomic motion of proteins in a manner dependent on the field strength, providing new insights into how electric fields influence key proteins in macrophages.^177^

Magnetic fields induce morphological changes in macrophages by altering ion flow or disturbing the cell membrane. Wosik et al.^178^ found that non-uniform magnetic fields cause significant elongation of macrophages and rearrange their actin cytoskeleton, Golgi complex, and TRPM2 cation channel receptors. These magnetic field-induced changes are similar to those resulting from RhoA pathway inhibition, affecting macrophage migration ability and the expression of molecular markers. Magnetic fields have also been shown to regulate inflammation and oxidative stress responses. During acute inflammatory reactions, exposure to time-varying magnetic fields significantly downregulates IL-6 and IL-10 levels in macrophages.^179^ Complex magnetic fields can reduce ROS production, promote the polarization of macrophages towards the M2 anti-inflammatory phenotype, and upregulate the expression of molecular markers associated with wound healing, which aids in the healing of diabetic foot ulcers.^180^ Additionally, pulsed electromagnetic fields promote the M2 phenotype of macrophages via the FAK signaling pathway, regulate the synthesis of anti-inflammatory mediators, and improve communication between macrophages and human tendon cells, supporting their role in tendon healing and inflammation regulation.^181^ Sun and colleagues^182^ found that hydroxyapatite scaffolds containing magnetic nanoparticles promote M2 polarization of macrophages by activating the PPAR signaling pathway and inhibiting the JAK-STAT signaling pathway. This process is accompanied by the synergistic inhibition of M1 macrophages by the external magnetic field, revealing the key role of magnetic fields in macrophage polarization.

Low-intensity pulsed ultrasound (LIPUS) and shockwave therapy have also been shown to activate macrophages, promoting their polarization toward the M2 phenotype and facilitating tissue regeneration by regulating apoptosis and inflammatory responses.^183,184^ Specifically, LIPUS can promote macrophage M2 polarization through the WNT signaling pathway while reducing necrosis-like apoptosis.^185^ Wilson and colleagues^186^ found that clinically used low-intensity shockwave therapy significantly reduced the overall number of macrophages in chronic ulcer tissues, but the proportion of M2 macrophages increased. These physical stimulation methods offer advantages such as low cost, ease of operation, and minimal side effects. Additionally, treatment can be applied locally to specific tissue areas, providing a potential adjunctive therapy for treating bone bacterial infections. However, it is necessary to balance the tissue penetration ability of these physical stimuli and their specific effects on macrophages at different intensities.

### Biomaterial surfaces and bacterial adhesion

#### Bacterial adhesion mechanisms on implant surfaces

Bacterial adhesion to the surface of implants is the initial and critical step in implant-associated infections. This process is complex and involves various chemical and physical interactions. During adhesion, bacteria undergo multiple stages, including initial attachment, cell proliferation, and biofilm formation. The initial reversible adhesion typically occurs when motile bacteria come sufficiently close to the implant surface, becoming temporarily fixed through physical forces such as Brownian motion, van der Waals forces, electrostatic interactions, and hydrophobic effects.^187^ Additionally, chemotaxis driven by concentration gradients of chemical inducers like amino acids and sugars may further promote the initiation and progression of this process.^188^ As the contact becomes closer, short-range interactions such as chemical and ionic bonding become particularly important, facilitating the transition from initial attachment to stable adhesion.^189^ Subsequently, with the interaction between bacterial cells and molecules with the surface, bacteria utilize specific adhesion factors (such as adhesins and biofilm matrix) to form stronger bonds with the surface. In terms of mechanism and kinetic modeling, bacteria exhibit adhesion behavior similar to colloidal particles at this stage. However, the actual adhesion outcome is influenced by the physicochemical properties and wettability of the material surface, as well as the heterogeneity of bacterial populations, making classical colloid theories (such as the XDLVO theory) insufficient to fully predict bacterial adhesion behavior.^190^ For example, *S. epidermidis* binds directly to inert surfaces such as polystyrene through the AtlE autolysin,^191^ whereas *S. aureus* preferentially recognizes host-protein-coated surfaces modified by host proteins (fibrinogen, fibronectin) via its AtlA autolysin.^192^ Additionally, bacterial appendages (such as nanofibers and pili) promote biofilm formation through nonspecific anchoring.^193^

Interestingly, when the implant surface is coated with host proteins, bacteria achieve irreversible adhesion through specific adhesins. *Staphylococci* utilize adhesins from the Microbial Surface Components Recognizing Adhesive Matrix Molecules (MSCRAMMs) family, such as ClfA and FnBPs, to specifically recognize matrix proteins like collagen and fibronectin.^194,195^ For example, the ability of *S. aureus* to bind collagen and bone sialoprotein may mediate the occurrence and development of bone infections,^196^ while *S. epidermidis* mediates catheter-related infections through surface molecules interacting with fibrinogen.^197^ Moreover, the binding mechanism of *S. epidermidis* to fibronectin involves recognition of the carboxyl-terminal binding domain.^198^ It is noteworthy that adhesins are multifunctional, with some members simultaneously mediating host immune modulation and bacterial invasion of host cells.^199^ Key molecules involved in different stages of adhesion, such as AtlE, AtlA, and MSCRAMMs, along with their target sites (collagen, fibronectin, etc.), form the molecular basis for bacterial adhesion and penetration of the implant surface barrier. This process may be regulated by both the characteristics of the implant and the host microenvironment.

Over time, bacteria fix themselves onto the surface by producing extracellular polymeric substances (EPS), entering a more stable biofilm phase.^200^ Biofilm formation can be summarized into five stages: attachment, colonization, microcolony formation, maturation, and dispersion followed by reformation.^201^ The continuous aggregation of bacteria and formation of microcolonies gradually constructs a mature biofilm with a 3D structure, which can withstand certain shear forces and provide support and protection for surrounding microorganisms. As specific enzymes participate in the degradation of the matrix, this leads to the detachment and dispersion of cells from the biofilm.^202^ However, this is not the endpoint; a new round of biofilm renewal helps bacteria adapt to various extreme environments and resist attacks from immune cells and antimicrobial agents. Moreover, the role of signal transduction mechanisms in the dynamic changes of bacterial adhesion cannot be overlooked. Bacteria sense external environmental changes and regulate internal signaling pathways, allowing for fine control of adhesion and biofilm formation. For example, bacteria can sense signals such as magnesium ions and low pH in the environment through the PhoP/PhoQ system, regulating the expression of adhesion-related genes, thereby enhancing their adhesion capacity and biofilm formation.^203,204^ The complexity of this signaling mechanism enables bacteria to rapidly adapt in fluctuating microenvironments, improving their ability to colonize biomaterial surfaces. The persistence and chronic nature of infections caused by bacterial biofilms pose challenges to traditional antimicrobial treatments. Therefore, exploring molecular targets during the initial bacterial adhesion phase on implant surfaces and carrying out proactive interventions may offer new strategies and approaches for combating biofilms, such as applying antimicrobial or immunomodulatory modifications to the implant surface.

#### Influence of biomaterial surface properties on bacterial adhesion

The “race for the surface” between host tissue cells and bacteria partially determines the likelihood of tissue integration or infection, with the surface characteristics of the substrate playing a pivotal role in the outcome. Similar to how material surface properties influence macrophage behavior, the interactions between bacteria and the surface are also affected by multiple factors, including surface topography, roughness, wettability, and charge. By rationally designing the surface properties of biomaterials, it is possible to effectively reduce bacterial adhesion and biofilm formation, thereby improving the clinical performance of implants in the treatment of osteomyelitis.

##### Surface topography

Bacteria are capable of sensing mechanical signals associated with surface topography, and such topographical features at both the microscale and nanoscale can significantly influence bacterial adhesion behavior. Generally, microscale surface features affect bacterial attachment through hydrodynamic mechanisms, whereas nanoscale features influence adhesion through chemical gradients, physicochemical forces, and deformation of the bacterial cell membrane.^205^

Recent advances in surface engineering have yielded promising results. Studies have shown that mimicking surface topographies found in nature can effectively enhance both the antibacterial properties and cytocompatibility of biomaterials. Examples include surfaces inspired by lotus leaves,^206^ sharks,^207^ cicadas,^208^ dragonfly wings,^209^ and butterflies.^210^ For instance, the microdenticle structures of shark skin slightly promote bacterial adhesion at the early stage but significantly inhibit biofilm formation over time.^207^ The micro/nanostructures of box-patterned gecko skin exhibit extremely low microbial adhesion while demonstrating bactericidal activity against Gram-negative bacteria and maintaining excellent biocompatibility.^211^ Additionally, engineered surfaces such as dynamic wrinkled patterns on PDMS have been shown to inhibit biofilm formation by Pseudomonas aeruginosa by up to 80 percent.^212^ Perera-Costa et al.^213^ fabricated various microtopographies on PDMS, including square and circular protrusions or depressions and parallel grooves. Their findings revealed that regardless of the surface’s hydrophobic or hydrophilic nature, bacterial adhesion was significantly reduced, highlighting the effectiveness of microtopographical design as a physical antibacterial strategy.

In addition to influencing bacterial adhesion, certain surface topographies of materials can exert bactericidal effects by disrupting bacterial cell membranes. For example, the nanoscale pillar structures on cicada wings (200 nm in height and 60 nm in diameter) can mechanically stretch and rupture bacterial membranes, leading to cell death. For Gram-positive bacteria, which possess higher rigidity, materials may enhance bacterial sensitivity by reducing internal pressure.^214^ Zinc oxide nanorods with nanotopographical surfaces have demonstrated strong bactericidal activity against adhered Pseudomonas aeruginosa and exhibited a bactericidal effect against *S. epidermidis* that was 30 times greater than that of the control group.^215^ Therefore, by modifying and optimizing surface topography, it is possible to effectively reduce bacterial adhesion and enhance the antimicrobial performance of biomaterials.

##### Surface roughness

Roughness is a key physical parameter of biomaterial surface properties and has a significant impact on bacterial adhesion behavior. Currently, the influence of surface roughness on bacterial adhesion remains a topic of debate. Increased surface roughness typically promotes bacterial adhesion and biofilm formation by expanding the effective contact area and providing physical barriers against shear forces.^216,217^ In contrast, smooth surfaces tend to reduce biofilm development. For instance, an increase in the surface roughness of zirconia materials has been associated with enhanced initial adhesion and attachment ability of *Streptococcus mutans*.^218^ Another study found a positive correlation between increased nanoscale roughness (from 29 to 214 nm) and bioadhesion, with biofilm accumulation being greater on irregular surfaces compared to flat ones.^219^ However, this positive correlation is not absolute. Some experiments have shown that once roughness exceeds a certain threshold, the adhesion rate may actually decrease. For example, Singh et al.^220^ observed that bacterial adhesion and biofilm formation increased on surfaces with specific nanoscale roughness (20 nm), but significantly decreased when the roughness reached 25 nm, thereby inhibiting biofilm development. This may be attributed to the enhanced protein adsorption induced by increased roughness, which forms an intermediate layer that indirectly suppresses direct contact between bacteria and the material surface.

Studies have also found that the selection of roughness parameters is crucial for the reliability of conclusions. Traditional parameters, such as average surface roughness (Ra) and root-mean-square surface roughness (Rrms), may lead to experimental biases as they cannot accurately describe the geometric distribution and morphological differences of surface features.^221^ For example, surfaces with entirely different structures may exhibit similar Ra and Rrms values. Therefore, researchers have proposed combining multidimensional parameters, such as peak density (Sds) and expanded area ratio (Sdr), to more comprehensively characterize surface roughness.^222^

Furthermore, bacterial adhesion responses to roughness exhibit species-specific and environment-dependent characteristics. *Streptococcus species* show enhanced adhesion on rough surfaces, while *S. epidermidis* does not demonstrate significant differences.^223^ At the same time, proteins present in the medium, such as fibronectin, can regulate adhesion behavior by altering the surface chemical state. For instance, fibronectin adsorption on nanoscale rough titanium surfaces enhances bacterial adhesion, thereby inhibiting bacterial attachment.^224^ These conflicting results suggest that the impact of surface roughness on bacterial adhesion should be analyzed in conjunction with material properties, bacterial species, and environmental factors, as a single roughness parameter cannot fully predict the risk of biofilm formation.

##### Surface wettability

Surface wettability, as a key physical property, is determined by both the surface energy and the microscopic roughness of the material.^225^ Generally, materials with high surface energy tend to enhance surface hydrophilicity, while materials with low surface energy suppress liquid spreading, reducing surface wettability.^226^ This property directly influences the interaction forces between bacteria and material surfaces, such as through the regulation of van der Waals forces, electrostatic forces, and acid-base interactions.^227^ Thermodynamic models indicate that bacterial adhesion tendency is related to the matching of surface free energy: hydrophobic bacteria preferentially adhere to hydrophobic materials, while hydrophilic bacteria prefer hydrophilic surfaces.^228^ The DLVO theory and its extensions further suggest that most bacteria (0.5–2 μm) act as colloidal particles, and their adhesion is regulated by surface distance and solution ionic strength.^229^

Additionally, extreme water contact angles, such as those on superhydrophobic and superhydrophilic surfaces, can significantly reduce bacterial adhesion. Superhydrophobic surfaces reduce the solid-liquid contact area by trapping an air layer with micro/nano surface structures (Cassie-Baxter state), thus reducing bacterial adhesion.^230^ Superhydrophilic surfaces, on the other hand, weaken the direct interaction between bacteria and the substrate through a dense water molecular layer.^231^ For instance, Ozkan et al.^232^ achieved superhydrophobic properties by constructing layered micro/nano structures on commercial polyurethane sponges, which led to a 99% reduction in the adhesion of *S. aureus* within 4 days. Furthermore, zwitterionic polymers are generally electrically neutral, which gives them superhydrophilic properties and strong resistance to bacterial adsorption and growth, demonstrating excellent application results in infectious wounds.^233^ Poly-4-hydroxybutyrate (P4HB) has been shown to be hydrophilic, significantly reducing the number of *S. aureus* and *Escherichia coli*.^234^ It is noteworthy that Lorenzetti et al.^235^ found that significantly hydrophilic TiO_2_-anatase coatings exhibited more initial bacterial attachment. There are inconsistent results regarding the effect of hydrophilic materials on bacterial adhesion, which may be related to differences in experimental systems, bacterial species characteristics, and environmental dynamic conditions.

##### Surface charge

The surface charge of biomaterials significantly influences bacterial adhesion, though its underlying mechanisms are complex and species-dependent.^236^ It is widely recognized that bacterial cells typically carry a net negative charge due to the presence of surface carboxyl, amino, and phosphate groups, making them more prone to adhere to positively charged surfaces.^237^ In an early study, the initial adhesion rate of Pseudomonas aeruginosa on positively charged polymethacrylate surfaces was found to be twice as high as that on negatively charged surfaces.^238^ This phenomenon has been confirmed in adhesion experiments involving *S. aureus* and *Escherichia coli* on various positively charged materials.^239,240^ However, further research has shown that the relationship between surface charge and bacterial adhesion is not entirely consistent. For example, positively charged surfaces can significantly inhibit adhesion of certain Gram-positive bacteria, such as *Streptococcus mutans*.^241^ Notably, some Gram-negative bacteria, such as *Pseudomonas aeruginosa* and *Escherichia coli*, can overcome electrostatic repulsion and adhere to negatively charged surfaces by means of surface appendages like pili or through the secretion of lipopolysaccharides.^242^ Studies have also indicated that while positively charged surfaces may enhance adhesion, materials containing cationic groups such as quaternary ammonium salts can reduce the number of viable bacteria through their antibacterial effects. This antimicrobial effect can be enhanced in dynamic systems, such as in oral materials, where mechanical forces facilitate the removal of dead cells, but may be diminished in static systems due to the accumulation of cellular debris.^243^ Moreover, the interaction between material surface charge and bacterial species is highly specific. For instance, positively or negatively charged polyelectrolyte multilayer films have been shown to selectively inhibit the adhesion of Gram-negative and Gram-positive bacteria, respectively.^244^

Interestingly, the impact of surface charge on biofilm development differs from its effect on initial bacterial adhesion. Although surfaces with high charge density may promote bacterial adhesion, they can simultaneously reduce cellular viability and inhibit subsequent biofilm maturation. The structure and mechanical strength of biofilms formed on surfaces with different charges also vary.^245^ For example, *Escherichia coli* biofilms on positively charged surfaces tend to be homogeneous and dense, exhibiting strong resistance to shear forces, whereas those on negatively charged surfaces are more likely to form heterogeneous, loose, mushroom-shaped structures. These conflicting phenomena suggest that the relationship between surface charge and bacterial adhesion must be systematically investigated in the context of material properties, bacterial species, and environmental conditions.

### Influence of environmental factors on bacterial adhesion

It is noteworthy that environmental factors play a critical role in bacterial adhesion and subsequent biofilm formation. Bacterial adhesion, as the initial step of biofilm development, is influenced by a variety of environmental conditions, including host serum proteins, fluid dynamics, temperature, pH, and the presence of antibiotics. Investigating the interactions between these factors and bacteria, along with the underlying mechanisms, is essential for the development of novel antibacterial adhesion-resistant implants and strategies.

#### Serum proteins

Albumin, upon adsorption to material surfaces, can significantly inhibit the adhesion of various bacteria such as *S. aureus* by increasing surface hydrophilicity or directly binding to bacterial cells.^246^ This inhibitory effect is shear rate-dependent, with higher shear forces further reducing adhesion rates. For instance, Dickinson et al.^247^ found that albumin coatings suppressed bacterial adhesion on polyurethane, aminated, and sulfonated polyurethane surfaces. Additionally, hydrophobic nanofiber membranes containing fluorocarbon chains have been shown to enhance albumin adsorption and thereby reduce bacterial adhesion.^248^ In contrast, fibrinogen promotes bacterial adhesion through ligand-receptor specific interactions with bacterial surface components such as staphylococcal surface ligands, and this effect is independent of shear rate.^249^ Thrombin facilitates the polymerization of fibrinogen into fibrin networks, enhancing platelet aggregation and thrombus stability, and thereby indirectly promoting bacterial adhesion. For example, when fibrinogen or platelets are pre-adsorbed onto polyurethane surfaces, a significant increase in *S. aureus* adhesion is observed.^250^

Additionally, the competitive adsorption of multiple proteins presents in plasma or serum, such as the Vroman effect, may alter the protein composition on material surfaces and influence bacterial adhesion. For example, high-molecular-weight proteins can displace fibrinogen, thereby reducing its adhesion-promoting effect on coagulase-negative *staphylococci* (CoNS).^251^ Moreover, material surface hydrophilicity (acid-base treatment of titanium) and surface roughness (porous structures) can modulate the thickness and composition of the adsorbed protein layer, indirectly suppressing bacterial adhesion.^252^ Recently, Ajdnik et al.^253^ developed a bioactive nanocoating that demonstrated excellent protein-repellent properties and antimicrobial performance. In summary, the effect of serum or tissue proteins on bacterial adhesion depends on the protein species, surface properties of the material, and bacterial receptor specificity. The underlying mechanisms involve direct binding, modulation of surface characteristics, and competitive adsorption.

#### Hydrodynamics

Hydrodynamic forces are recognized as critical factors influencing bacterial adhesion and biofilm formation,^254^ involving shear stress effects, dynamic biofilm regulation, and the surface microenvironment. Increased shear rates generally enhance the detachment effect of fluid flow, significantly reducing bacterial adhesion on most material surfaces.^255^ Katsikogianni et al.^256^ found that when the shear rate increased from 150 s⁻¹ to 1 500 s⁻¹, the number of adherent bacteria on various surfaces generally decreased, potentially due to the formation of mechanical shelters on rough surfaces. Moreover, bacterial adhesion efficiency exhibits an optimal flow rate range. Mohamed et al.^257^ reported that the adhesion of *S. aureus* increased at shear rates between 50 and 300 s⁻¹, but significantly declined above 500 s⁻¹. This effect is regulated by the density of receptors on the bacterial cell surface.

Shear flow can promote biofilm formation and enhance mechanical properties by inducing the synthesis of EPS. For example, Hou et al.^258^ demonstrated that under dynamic conditions, *S. aureus* exhibited increased EPS production, resulting in a matrix structure with greater compressive resistance. Multispecies biofilms tend to exhibit higher elastic moduli in flowing environments; for instance, Paramonova et al.^259^ observed that dual-species biofilms had tenfold greater compressive strength compared to monospecies biofilms. However, excessive shear stress may disrupt the adhesion between bacteria and the surface through vortex effects, leading to the formation of loosely structured biofilms. Song et al.^260^ found that biofilms formed under dynamic culture conditions were looser and contained fewer bacterial cells.

Additionally, microtopographical surface features can influence adhesion distribution by altering the local flow field. Lee et al.^261^ found through simulations that the shear stress at the tips of prism-shaped microstructures was higher than in the grooves, leading to preferential bacterial deposition in low-shear regions. Hydrophobic surfaces exhibit enhanced anti-adhesion properties under flow conditions; Hizal et al.^262^ demonstrated that nanostructured hydrophobic materials retained an air layer that reduced the contact area with bacterial suspension, thereby improving the efficiency of fluid-mediated removal. Therefore, when evaluating anti-adhesive materials or infection mechanisms, it is essential to consider the effects of dynamic fluid environments comprehensively.

#### Other factors

Changes in environmental pH significantly affect bacterial adhesion. Bacteria adapt to intracellular and extracellular pH fluctuations by regulating the activity and synthesis of membrane proteins,^263^ but the secretion of extracellular polymers such as polysaccharides is more sensitive to pH changes. For most bacterial species, the optimal pH for secretion is around 7.^264,265^ A previous study demonstrated that *S. aureus* ATCC 25923 exhibits the strongest adhesion to glass surfaces at pH 4 to 6, while its adhesion weakens under extremely acidic (pH = 2–3) or alkaline conditions, with cells tending to form aggregates.^266^ In a study on bone implant-associated infections, Kinnari et al.^267^ found that when the pH of the bone tissue microenvironment drops to 6.8 due to trauma or infection, the adhesion of *S. aureus* and *S. epidermidis* to hydroxyapatite and biphasic calcium phosphate is significantly reduced. This may be attributed to the limited porosity of the two materials, which ensures that their clinical performance remains unaffected.

Antibiotics can reduce infection risk by diminishing bacterial surface adhesion or inhibiting early biofilm formation. For instance, a previous study demonstrated that a biomimetic coating containing tobramycin on titanium alloy surfaces exhibited a concentration-dependent inhibitory effect against *S. aureus*.^268^ Notably, bacteria within biofilms can exhibit antibiotic resistance up to 1 000 times higher than planktonic bacteria, due to the protective extracellular matrix and metabolic heterogeneity.^269^ Cerca et al.^270^ found that combinations of antibiotics at sub-minimum inhibitory concentrations, such as vancomycin and cefazolin, suppressed the adhesion of CoNS on acrylic surfaces by more than 70%, though their impact on established biofilms was limited. Another study showed that prophylactic antibiotic application, such as linezolid, was most effective within 6 h prior to bacterial adhesion, with delayed administration resulting in a marked reduction in efficacy.^271^ These findings indicate that early intervention targeting bacteria adhering to implant surfaces is a key strategy for reducing the recurrence of bone infections.

Additionally, bacteria exhibit stronger adhesion capabilities at higher growth temperatures, such as 37 °C. For instance, *Pseudomonas aeruginosa* and *S. aureus* have been shown to adhere significantly more to surfaces like stainless steel and polycarbonate after incubation at 37 °C compared to strains cultured at lower temperatures (20 °C or 15 °C).^272^ The optimal adhesion temperature for *Legionella* has also been identified as 36 °C.^273^ Although temperature plays a significant role in regulating adhesion, its effects often act in conjunction with surface roughness or the chemical properties of the material.

### “Race for the surface” between host cells and bacteria

The “race for the surface” between host cells and bacteria on the surface of biomaterials is a complex and dynamic process involving interactions between host cells and pathogens. Current consensus suggests that rapid integration of biomaterials with host tissues and cells after implantation is crucial to preventing bacterial adhesion and biofilm formation.^274^ Specifically, if host cells and tissues achieve rapid and successful integration with the implant and occupy its surface, while host immune cells, such as macrophages, neutrophils, T cells, and B cells, exert various synergistic bactericidal effects, bacterial attachment and colonization on the implant surface can be significantly reduced, ultimately achieving a favorable outcome. From the perspective of microbial invasion, if bacteria manage to colonize the surface and form a biofilm before the host cells integrate with the implant, through mechanisms of interference and immune evasion, it becomes extremely difficult to eradicate the infection, often necessitating thorough debridement and implant revision surgery.^275^ To ensure host cells take the lead in the “race for the surface”, two intuitive strategies are proposed. First, thorough debridement of osteomyelitis tissue prior to implantation, combined with systemic or local antibiotic therapy, is essential to fully control infection and provide a favorable microenvironment for osseointegration, and this represents the clinical “gold standard”. Second, in-depth investigations into the interactions between host tissue cells and bacteria with biomaterials should be conducted, with a focus on surface modification strategies that give host cells the advantage in the “race for the surface”. From a materials science perspective, surface modifications that endow the implant with direct antibacterial properties, or enhance rapid osseointegration, or activate immune-mediated bactericidal responses represent promising approaches to achieving this goal.

However, as previously mentioned, the physicochemical properties of material surfaces exert complex and multifaceted effects on both host cells and bacteria. Their regulatory roles in macrophage polarization, osteogenic cell activity, and bacterial behavior are often contradictory. For instance, in terms of surface roughness, moderate roughness or specific nanoscale topographies can promote M2 polarization, thereby driving anti-inflammatory responses and bone regeneration.^108,109^ However, micro/nanostructured rough surfaces generally facilitate initial bacterial adhesion by increasing contact area and creating physical barriers,^218^ leading to uncertainty over whether bacterial or host cell colonization will dominate. Regarding surface wettability, hydrophilic materials can enhance M2 macrophage polarization,^119,120^ but extremely hydrophilic surfaces may also favor initial bacterial adhesion.^235^ As for surface charge, cationic surfaces may boost anti-inflammatory M2 phenotype while simultaneously raising the risk of pathogenic bacterial attachment.^130,238,240^ In summary, the requirements for surface characteristics in the “race for the surface” between microbes and host cells are inherently complex and variable.

**Fig. 4:**
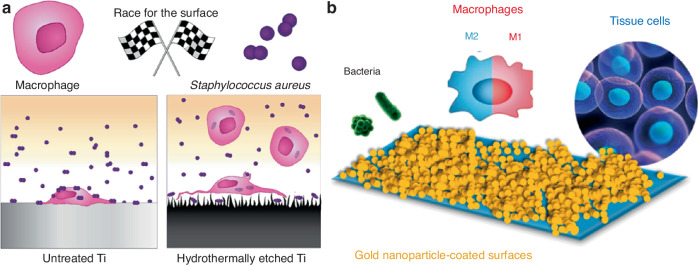
Existing in vitro co-culture systems based on the “race for the surface” concept. **a** *Staphylococcus aureus* and macrophages were co-cultured on different titanium surfaces; compared to untreated titanium, macrophages dominated on surfaces with sharp features, winning the “race for the surface”. Reproduced with permission from ref. ^276^ Copyright 2023, American Chemical Society. **b** A triple-culture system established on gold nanoparticle-coated surfaces, involving macrophages, tissue cells, and bacteria, was designed to investigate the in vitro “race for the surface” between bacteria and host cells. Reproduced with permission from ref. ^277^ Copyright 2020, American Chemical Society

Recently, Bright et al.^276^ co-cultured bacteria with macrophages and found that on titanium surfaces with sharp features, macrophages dominated, whereas on untreated titanium surfaces, bacteria completely overwhelmed the macrophages (Fig. 4a). Differential gene expression analysis revealed that the reduced rate of peptidoglycan biosynthesis on sharp-featured surfaces laid the foundation for the competitive advantage of macrophages. Luan et al.^277^ developed a triple-culture system on gold nanoparticle-coated surfaces involving macrophages, tissue cells (U2OS or hMSCs), and bacteria (Fig. 4b). In this system, macrophages exhibited an anti-inflammatory M2 phenotype and facilitated tissue cell repair, promoting surface coverage and adhesion of host cells. However, this beneficial effect was most pronounced under contamination with Gram-positive *S. aureus*, and was not observed under *Escherichia coli* exposure. Therefore, the success of tissue cells in the “race for the surface” largely depends on factors such as surface topography, roughness, wettability, and bacterial species. Based on these findings, we envision an ideal scenario in which material surfaces can precisely and dynamically regulate macrophage polarization and immune activation responses: when bacteria approach the surface, the material would exhibit inherent bactericidal properties and induce pro-inflammatory M1 polarization to eliminate the infection; when tissue cells approach, the surface would enhance adhesion, induce M2 polarization, and promote tissue regeneration and osseointegration, with synergistic regulation from environmental cues. Given the limitations of in vitro experiments in elucidating bacterial adhesion and biofilm formation on implant surfaces, exploring the “race for the surface” through in vivo model systems is critical for uncovering underlying mechanisms and advancing the design and development of immunomodulatory biomaterials.

## Biomaterials modulate macrophage polarization phenotypes

Over the past decade, with significant advances in the field of osteoimmunology, it has become widely recognized that biomaterials play a critical role in regulating the fate of immune cells after implantation. In bone infectious diseases, including implant-related infections, trauma-related infections, and osteomyelitis, understanding the immune response to biomaterial implantation is crucial. As the primary effector cells in immune responses, macrophages are involved in the entire regulatory process of pathogen clearance and bone formation promotion during bone infections through their phenotype switching. However, macrophages do not exist solely in the simple M1/M2 states, and changes in the infection microenvironment may push them into intermediate states between M1 and M2. In acute infections, inducing the M2 phenotype in macrophages to promote tissue repair while preventing rapid accumulation of inflammatory factors is a common strategy. In chronic infections, it is critical for biomaterials to promote M1 macrophage polarization to enhance the clearance of pathogens, biofilms, and intracellular infections. Therefore, given the dynamic changes in macrophage phenotypes at different stages of infection, this section focuses on current biomaterial strategies for anti-inflammatory, pro-inflammatory, and sequential regulation of macrophages in the context of acute and chronic bone infections. These strategies provide a reference for stratified and staged treatments of bone infections.

### Acute infections: inducing anti-inflammatory macrophage phenotypes to improve bone tissue regeneration

In the acute phase of bacterial infection, immune cells play a crucial role in pathogen clearance, but an excessive release of pro-inflammatory cytokines can lead to a dangerous cytokine storm. This immune overreaction disrupts the delicate balance of the immune microenvironment and can cause significant tissue damage.^278^ Macrophages, a key component of this immune response, are highly versatile cells capable of shifting between a pro-inflammatory M1 state and an anti-inflammatory M2 state based on environmental signals. Harnessing this plasticity has become a focal point in the development of immunomodulatory biomaterials.^279^ These advanced materials aim to guide macrophages towards an anti-inflammatory state, which not only helps to prevent cytokine storms but also accelerates bone tissue healing, offering a promising approach to treating skeletal bacterial infections and restoring immune homeostasis (Table 3).

**Table 3:** Summary of advanced biomaterials for modulating macrophage anti-inflammatory polarization in acute bone infections

Types of biomaterials	Major components	In vitro model	In vivo model	Macrophage polarization	Functions	Ref.
Titanium-based biomaterials	Ti, antimicrobial peptide (HHC36), MSNs	RAW 264.7 macrophages, mouse BMSCs	Rabbit model of infection bone defect	M2 polarization	In vitro: (1) Excellent antibacterial performance; (2) The biomaterial induces M2 macrophage polarization to activate anti-inflammatory responses; (3) Promotes osteogenic activity and exhibits good biocompatibility. In vivo: (1) It inhibits bacterial infections; (2) Induces M2 macrophage polarization for anti-inflammatory effects and enhances bone integration.	^285^
	Ti, ZIF-8, honeycomb-like TiO2 nanotube array (TNT)	RAW264.7 cells and BMSCs	Rat implant-associated infection model	M2 polarization	In vitro: (1) The glucose oxidase activity of ZIF-8 promotes antibacterial effects; (2) Zn²⁺ release hydrolyzes extracellular DNA, enhancing bacterial killing and preventing biofilm formation; (3) It promotes osteogenic differentiation; (4) Induces macrophage M2 polarization and inhibits the overexpression of pro-inflammatory factors. In vivo: (1) Bacterial eradication; (2) It induces M2 macrophage polarization and promotes bone integration.	^286^
	Ti, hydrogel coating composed of konjac gum and gelatin, tannic acid-d-tyrosine nanoparticles	RAW264.7 cells and MSCs	Rat implant-associated infection model	M2 polarization	In vitro: (1) Excellent photothermal effects synergize to kill bacteria and eliminate biofilms; (2) The biomaterial removes excessive intracellular ROS and induces macrophage M2 polarization, reducing pro-inflammatory responses; (3) Promotes MSC proliferation and osteogenic differentiation. In vivo: (1) Bacterial eradication; (2) It induces M2 macrophage polarization, alleviates inflammatory responses, and promotes bone integration.	^287^
	Ti, bioactive polydopamine/Ti3C2/poly(vinylidene fluoride trifluoroethylene) (PDA/Ti3C2/P(VDF-TrFE)) nanocomposite	RAW264.7 cells and MSCs	/	M2 polarization	In vitro: (1) The biomaterial directly promotes the spreading, growth, and differentiation of BMSCs; (2) Thermal stimulation enhances HSP47 synthesis and activates the MEK/ERK pathway to promote osteogenic differentiation; (3) Mild thermal stimulation induces macrophage M2 polarization, reducing inflammatory responses; (4) Excellent antibacterial properties.	^288^
	Ti6Al4V-6Cu alloy	RAW264.7 cells, HGFs, HUVECs, and osteoblasts	/	M2 polarization	In vitro: (1) The biomaterial promotes angiogenesis in HUVECs; (2) Induces macrophage M2 polarization, reducing inflammatory responses; (3) Excellent biocompatibility.	^289^
	Ti6Al4V-6Cu alloy	Osteoporotic macrophages	/	M2 polarization	In vitro: The biomaterial induces M2 polarization of osteoporotic macrophages in an infected microenvironment, promoting the release of anti-inflammatory factors.	^290^
PEEK-based biomaterials	PEEK, polydopamine-bioactive glass nanoparticles	RAW264.7 cells, BMSC, MC3T3-E1 cells, and ADSCs	Rat model of bone defect	M2 polarization	In vitro: (1) Excellent photothermal antibacterial activity; (2) The biomaterial promotes osteogenic differentiation of BSMCs; (3) Induces macrophage M2 polarization and reduces the expression of inflammatory factors. In vivo: It induces macrophage M2 polarization and promotes bone integration.	^294^
	PEEK, PDA, GS layer	MC3T3-E1 and RAW264.7 cells	Rat implant-associated infection model	M2 polarization	In vitro: (1) Excellent activity in promoting osteogenic differentiation; (2) The biomaterial induces macrophage M2 polarization; (3) Exhibits excellent antibacterial properties. In vivo: It induces macrophage M2 polarization and promotes bone integration.	^295^
	3D porous sulfonated PEEK, sodium butyrate	RAW264.7 cells and rat BMSCs	Rat osteomyelitis model	M2 polarization	In vitro: (1) Enhanced macrophage phagocytic activity and elevated ROS levels promote bacterial eradication; (2) The biomaterial induces macrophage M2 polarization and stimulates the secretion of anti-inflammatory cytokines; (3) Exhibits excellent osteogenic activity. In vivo: (1) Excellent anti-infection capability; (2) Good bone repair capability.	^296^
Metal/metal oxide NPs	Ti, Ag NPs, Sr	MC3T3-E1 cells and RAW264.7 cells	Rat model of infected femoral metaphysis	M2 polarization	In vitro: (1) The biomaterial eliminates pathogens through the release of Ag⁺ and Sr²⁺ and promotes osteoblast. differentiation; (2) It induces macrophage M2 polarization. In vivo: Enhanced bone integration performance.	^300^
	50 nm Au NPs	RAW264.7 cells and BMSCs	Rat osteomyelitis model	M2 polarization	In vitro: (1) The biomaterial induces macrophage M2 polarization by inhibiting the NF-κB signaling pathway; (2) It promotes osteogenic differentiation of BSMCs; (3) Overexpression of TREM2 enhances macrophage phagocytosis of *Staphylococcus aureu*s. In vivo: It suppresses inflammatory responses, and promotes tissue repair.	^303^
	Lipopolysaccharide-treated macrophage cell membranes, AuNC	RAW264.7 cells	Mice model of bone defect	M2 polarization	In vitro: (1) Nanoparticles synergize with photodynamic therapy for antibacterial effects and remove residual ROS; (2) It induces macrophage M2 polarization In vivo: It induces macrophage M2 polarization, suppresses inflammatory responses, and promotes bone repair.	^304^
	CeO_2_ nanoceria, Ce6	RAW264.7 cells and MC3T3-E1cells	Rat models of periodontal inflammation	M2 polarization	In vitro: The biomaterial promotes macrophage M2 polarization and enhances anti-inflammatory responses. In vivo: It induces macrophage M2 polarization, suppresses inflammatory responses, and promotes tissue repair.	^307^
	ZnO nanowires, collagen fibrils	RAW264.7 cells and BMSCs	Rat model of infectious mandibular defect	M2 polarization	In vitro: (1) The biomaterial promotes BMSC adhesion, proliferation, and osteogenic differentiation; (2) It releases Zn²⁺ to inhibit bacterial activity. In vivo: It induces macrophage M2 polarization, suppresses inflammatory responses, and promotes the healing of infected bone defects.	^308^
	Borosilicate bioactive glass (BSG) combined with ferroferric oxide (Fe_3_O_4_)	RAW264.7 cells and MSCs	Rabbit model of implant-related *S. aureus* bone infection	M2 polarization	In vitro: (1) The biomaterial promotes MSC osteogenic differentiation and mineralization; (2) Induces macrophage M2 polarization and upregulates the expression of anti-inflammatory factors. In vivo: It completely eradicates bacteria while facilitating new bone formation.	^309^
Nanocomposites	Chitosan, puerarin	rBMSCs	Air pouch model, and rat model of femoral osteomyelitis	M2 polarization	In vitro: (1) Excellent antibacterial properties; (2) It rBMSC proliferation and osteogenic differentiation. In vivo: (1) The biomaterial removes LPS and regulates macrophage M2 polarization; (2) It improves bacterial infection-induced bone destruction.	^317^
	Biomimetic nanocomposites enabled loading of antibiotics (gentamicin and doxycycline)	hMSCs and THP-1	/	M2 polarization	In vitro: (1) Excellent antibacterial properties; (2) The biomaterial facilitates hMSC adhesion and osteoinduction; (3) Promotes macrophage M2 polarization	^318^
	ZIF-8, BPNs, GelMA, HAMA	MC3T3-E1 cells and RAW 264.7 cells	Rat model of severe cranial defect	M2 polarization	In vitro: (1) The biomaterial promotes macrophage M2 polarization and inhibits inflammatory responses; (2) Enhances osteogenic differentiation; (3) Photothermal antibacterial activity. In vivo: It achieves multiple functions, including antibacterial, anti-inflammatory, and bone regeneration promotion.	^319^

*ADSC* Adipose-derived stem cells, *Ag NPs* Silver nanoparticles, *AuNC* Gold nanocage, *Au NPs* Gold nanoparticles, *BMSCs* Bone marrow-derived mesenchymal stem cells, *BPNs* Black phosphorus nanosheets, *Ce6* Chlorin e6, *GelMA* Gelatin methacryloyl, *GS* Gentamicin sulfate, *HAMA* Hyaluronic Acid Methacryloyl, *HGFs* Human gingival fibroblasts, *HSP47* Heat shock protein 47, *HUVECs* Human umbilical vein endothelial cells, *MEK* Mitogen-activated protein kinase kinase, *MSNs* Mesoporous silica nanoparticles, *PDA* Polydopamine, *PEEK* Polyether ether ketone, *ROS* Reactive oxygen species, *TNT* Titanium nanotube array, *TREM2* Triggering receptor expressed on myeloid cells 2, *ZnO* Zinc oxide, *ZIF-8* Zeolitic imidazolate framework

#### Titanium-based biomaterials

Titanium-based biomaterials have demonstrated significant potential in the treatment of bone infectious diseases due to their excellent mechanical properties and biocompatibility, making them a suitable choice for bone implants.^280^ However, several studies have indicated that singular titanium implants may enhance pro-inflammatory responses and inflammatory storms by inducing M1 macrophage phenotypes, which are detrimental to the growth and healing of peri-implant bone tissue.^281,282^ Consequently, regulating the transition of macrophages between anti-inflammatory (M2) and pro-inflammatory (M1) phenotypes through surface modification of titanium-based materials (such as specific topographies and physicochemical surface modifications) has become a key therapeutic strategy for managing bone infections. Pitchai et al.^283^ summarized the effects of surface modifications on titanium-based materials over the past 30 years regarding macrophage activation and phenotype regulation in vitro. They found that titanium-based materials with rough and hydrophilic surfaces not only promote macrophage adhesion but also induce the formation of M2 macrophages, accompanied by the upregulation of anti-inflammatory cytokines. This provides valuable insights into the treatment of acute bacterial bone infections.

**Fig. 5:**
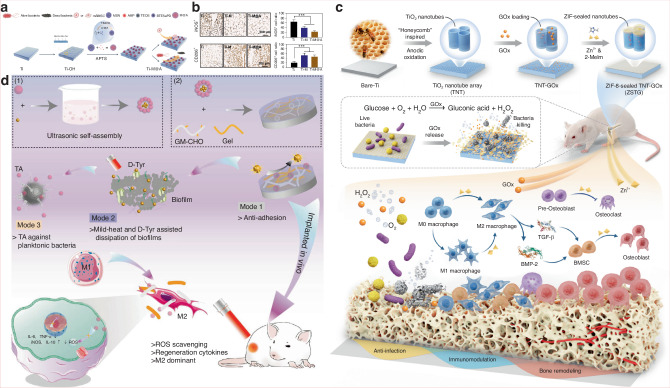
Ti-based biomaterials promoting bone tissue repair and regeneration by inducing M2 macrophage polarization. **a** Schematic illustration of the preparation of the functionalized titanium implant (Ti-M@A). **b** Ti-M@A implantation significantly reduced the M1 macrophage marker iNOS and upregulated the M2 macrophage marker CD206 after 60 days of treating infectious bone defects (scale = 200 μm). Reproduced with permission from ref. ^285^ Copyright 2024, Elsevier. **c** Preparation flowchart of the ZIF-8 smart drug delivery system based on the Ti substrate and its mechanism for treating implant-associated infections by modulating M2 macrophage polarization. Reproduced with permission from ref. ^286^ Copyright 2024, Elsevier. **d** Schematic illustration of the synthesis strategy and mechanism of functionalized Ti loaded with tannic acid-d-tyrosine nanoparticle hydrogels. Reproduced with permission from ref. ^287^ Copyright 2023, Wiley-VCH

Modifying the titanium surface with antimicrobial peptides, nanoparticles, and enzymes can not only effectively inhibit the growth of various pathogens but also modulate the immune microenvironment, promoting the polarization of macrophages toward the anti-inflammatory M2 phenotype. This reduces acute inflammatory responses and accelerates bone tissue repair.^284^ Wang et al.^285^ successfully modified titanium implants with mesoporous silica nanoparticles loaded with antimicrobial peptides, achieving both ROS and bacterial clearance. Simultaneously, this system effectively promoted the polarization of macrophages toward the anti-inflammatory M2 phenotype in vitro, as evidenced by the upregulation of the M2 marker CD206 (Fig. 5a, b). In an infectious bone defect model, the system exhibited consistent antibacterial effects and facilitated macrophage polarization toward the M2 phenotype (with a significant increase in CD206-positive macrophages), thereby accelerating osseointegration. Dong et al.^286^ developed a smart drug delivery system based on ZIF-8-modified titanium surfaces, which demonstrated excellent antibacterial and biofilm-inhibitory activities. Notably, the ZIF-sealed surfaces enhanced M2 macrophage polarization under lipopolysaccharide (LPS)-stimulated infectious microenvironments, thereby promoting osteogenic differentiation (Fig. 5c). In an implant-associated infections (IAIs) model, the system exhibited strong antibacterial performance and induced M2 macrophage polarization (evidenced by an increase in Arg-1-positive cells), ultimately facilitating osseointegration. Additionally, coating titanium surfaces with a hydrogel containing tannic acid-D-tyrosine-loaded nanoparticles can induce macrophage polarization toward the M2 phenotype and reduce inflammatory responses. Consistent with in vitro models, the femoral infection model showed a significant increase in CD206- and IL-10-positive macrophages and a marked decrease in CD86- and IL-6-positive macrophages in the hydrogel-coated group, demonstrating the capability of implant to reprogram macrophages toward the M2 phenotype^287^ (Fig. 5d). Xia et al.^288^ achieved the regulation of M2 macrophage polarization by modifying the titanium alloy surface with a polydopamine-coated Ti_3_C_2_ nanocoating, which not only reduced the inflammatory response in bone tissue but also promoted osteogenic differentiation through the MEK/ERK signaling pathway.

Beyond surface modification strategies, altering the composition of titanium alloys has also shown potential in promoting M2 macrophage polarization. For instance, Xu and colleagues^289^ utilized selective laser melting technology to synthesize copper-containing titanium alloys (Ti6Al4V-6Cu), which suppressed the inflammatory response of macrophages while enhancing vascularization in HUVECs. Further study in bone infection microenvironments revealed that the Ti6Al4V-6Cu alloy successfully inhibited M1 polarization induced by pathogenic bacterial LPS under acute infection conditions in vitro, facilitating the transition of macrophages to the M2 anti-inflammatory phenotype.^290^ This transition not only helps to inhibit the excessive release of pro-inflammatory factors but also accelerates the regeneration process of bone tissue.

#### Polyether ether ketone (PEEK)-based biomaterials

**Fig. 6:**
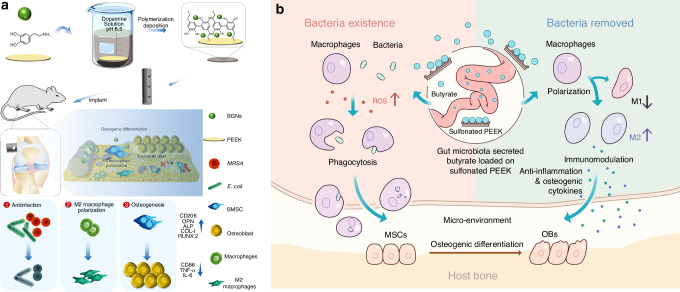
PEEK-based biomaterials promoting bone tissue repair by inducing M2 macrophage polarization. **a** Preparation process and functional schematic of multifunctional PEEK implants modified with polydopamine-bioactive glass nanoparticles (PDA-BGNs). Reproduced with permission from ref. ^294^ Copyright 2023, Wiley-VCH. **b** Schematic illustration of sodium butyrate-anchored SPEEK inducing M2 macrophage polarization to promote the osteogenesis process in a bacterial infection environment. Reproduced with permission from ref. ^296^ Copyright 2019, Royal Society of Chemistry

PEEK is a high-performance thermoplastic known for its exceptional mechanical strength, chemical resistance, and stability under harsh conditions. These characteristics make PEEK a highly attractive material for use in orthopedic and dental implants, particularly for bone tissue repair.^291^ However, bacterial infections at the implantation site and impaired immune function limit its broader application.^292^ Similar to titanium alloys, researchers have explored surface modification techniques to enhance antimicrobial and osteogenic activity of PEEK, as well as its ability to regulate macrophage polarization.^293^ Ma et al.^294^ functionalized PEEK implants by modifying the surface with polydopamine-bioactive glass nanoparticles (PDA-BGNs), enabling the material to provide multifunctional benefits, including anti-infection, osteoinduction, and immunomodulation (Fig. 6a). Specifically, this material induced macrophage polarization toward the M2 phenotype and promoted the osteogenic differentiation of BMSCs. These functions were validated in bone defect models, as evidenced by the upregulation of CD206 protein at the lesion sites and enhanced osseointegration. Another study employed dopamine self-polymerization to coat porous PEEK surfaces with gentamicin sulfate (GS), maintaining excellent antibacterial and osseointegration properties. Consistently, both in vitro and in vivo, the system induced polarization of anti-inflammatory M2 macrophages, thereby promoting bone regeneration in infectious bone defect.^295^ Additionally, loading sodium butyrate, a fermentation product of gut microbiota, onto three-dimensional porous sulfonated PEEK surfaces achieved M2 macrophage polarization and anti-inflammatory cytokine secretion in vitro. Furthermore, implantation in a rat osteomyelitis model confirmed its favorable bone regeneration performance^296^ (Fig. 6b). In summary, surface modifications of porous titanium alloys and PEEK during acute infections show great potential by inducing the M2 macrophage phenotype, thus playing anti-inflammatory and pro-regenerative roles. However, precisely and sustainably regulating these effects remains a challenge to be addressed. For instance, how to enable materials to rapidly and continuously modulate the M2 anti-inflammatory phenotype in the acute bone infection microenvironment.

#### Metals and oxides

**Fig. 7:**
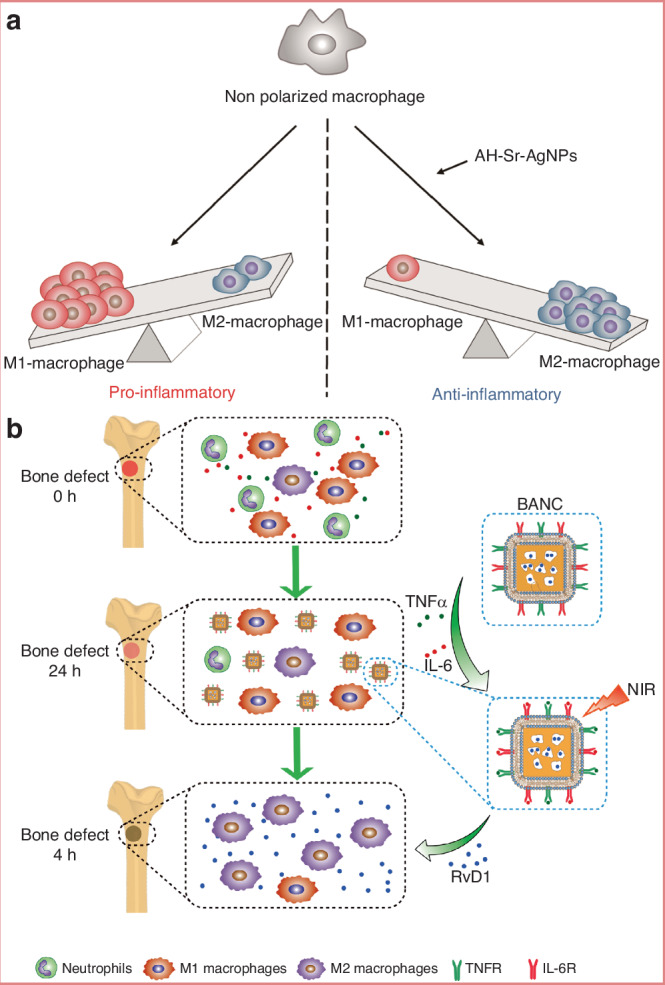
Regulation of M2 macrophage polarization by metal nanoparticle (NP)-based biomaterials for bone infection treatment. **a** Flow cytometry analysis showing enhanced M2 macrophage polarization induced by the AH-Sr-AgNPs delivery system in vitro. Reproduced with permission from ref. ^300^ Copyright 2019, Wiley-VCH. **b** Biomimetic anti-inflammatory nanocapsules (BANC) based on gold nanocages (AuNCs) promote bone tissue repair by inducing M2 macrophage polarization and suppressing the M1 phenotype.^304^ Copyright 2020, Elsevier

Metal/metal oxide nanoparticles (NPs) are widely applied in research fields such as antimicrobial and tissue regeneration. They can also interact with macrophages to regulate inflammation and regeneration responses.^297^ Based on the types and functions of metals, metal nanoparticles can trigger pro-inflammatory/anti-inflammatory polarization of macrophages, which is used for the integrated treatment of bone infections. Previous studies have reported that AgNPs are potential materials with antibacterial, osteogenic, and anti-inflammatory properties, showing good efficacy in treating osteomyelitis models in vivo.^298,299^ Li et al.^300^ synthesized a metal NP delivery system on the surface of titanium alloys, which releases Ag^+^ and Sr^2+^ ions to kill pathogens, regulate anti-inflammatory (M2) polarization of macrophages, and further promote the differentiation of osteogenic precursor cells in vitro (Fig. 7a). In a rabbit model of infectious bone defects, the biomaterial further showed good bone repair effects.

AuNPs have regulatory effects on both macrophage and osteoblast functions.^301^ It has been shown that 45 nm AuNPs can significantly suppress the inflammatory response induced by LPS and modulate macrophage polarization towards the M2 anti-inflammatory phenotype, thereby providing a favorable microenvironment for tissue repair.^302^ In the treatment of osteomyelitis, Wang et al.^303^ found that 50 nm gold nanoparticles (AuNPs) induced M2 macrophage polarization by inhibiting the NF-κB signaling pathway, thereby promoting the osteogenic differentiation of BMSCs in vitro. Owing to these mechanisms, the 50 nm AuNPs significantly reduced inflammation and promoted tissue repair in an osteomyelitis treatment model. Another study developed a biomimetic anti-inflammatory nanocapsule based on gold nanocages (AuNCs), which promoted M2 macrophage polarization and significantly improved bone tissue repair in both in vitro and in vivo models, providing a potential therapeutic strategy for the treatment of infectious bone diseases^304^ (Fig. 7b).

**Fig. 8:**
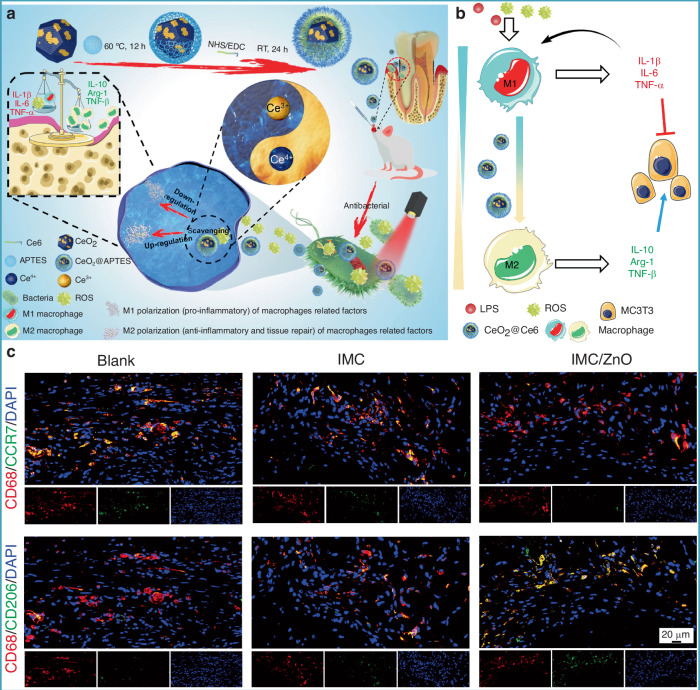
Regulation of macrophage polarization by metal oxide NP-based biomaterials for bone infection treatment. **a** Schematic illustration of the synthesis and mechanism of action of the CeO_2_@Ce6 nanocomposite. **b** Illustration showing the regulation of macrophage polarization by the CeO_2_@Ce6 nanocomposite and its effects on the expression of M1 and M2 macrophage markers. Reproduced with permission from ref. ^307^ Copyright 2021, Elsevier. **c** IMC/ZnO composite scaffold suppressing the expression of the M1 macrophage marker CCR7, while promoting the expression of the M2 macrophage marker CD206 after 2 weeks of implantation in an infectious bone defect model (scale = 20 μm). Reproduced with permission from ref. ^308^ Copyright 2024, Wiley-VCH

Additionally, various metal oxide NPs have been shown to stimulate M2 anti-inflammatory properties in macrophages, reducing the secretion of pro-inflammatory cytokines.^305^ CeO_2_ NPs, a multifunctional material with antioxidants, anti-inflammatory, and free radical-scavenging properties, have attracted widespread attention in the field of infection control.^306^ Sun et al.^307^ developed a multifunctional nanocomposite containing CeO_2_ NPs, which modulates the host immune response by scavenging excessive ROS and enhances the polarization of M2 macrophages (anti-inflammatory and regenerative) in vitro. In an animal model of periodontal infection, the material suppressed inflammation and promoted the repair of infected bone tissue by upregulating the expression of M2 phenotype markers (Fig. 8a, b). Given that ZnO NPs are potential broad-spectrum antibacterial agents, Zhang et al.^308^ synthesized an immunomodulatory scaffold through the co-assembly of collagen fibers and ZnO NPs and intrafibrillar mineralization. Animal results demonstrated that the scaffold released zinc ions within the infectious microenvironment, exerting antibacterial activity while simultaneously regulating M2 macrophage polarization, thereby promoting rapid healing of infectious mandibular defects (Fig. 8c). Additionally, a magnetic scaffold made of Fe_3_O_4,_ and borosilicate bioactive glass was shown to induce macrophage polarization towards the M2 phenotype in vitro, accompanied by the upregulation of anti-inflammatory genes (TGF-β1 and IL-1ra) and the downregulation of pro-inflammatory genes (IL-6 and IL-1β). In a rabbit model of infectious bone defects, the scaffold maintained good antibacterial properties and bone repair efficacy even after 42 days of implantation.^309^ Notably, certain FDA-approved nanomedicines, which regulate macrophage polarization towards the M1 phenotype, have made positive progress in anti-tumor therapy.^310^ This fact makes the immunomodulatory role of metal nanoparticles in macrophages a promising research direction in the field of bone infection.

#### Nanocomposites

**Fig. 9:**
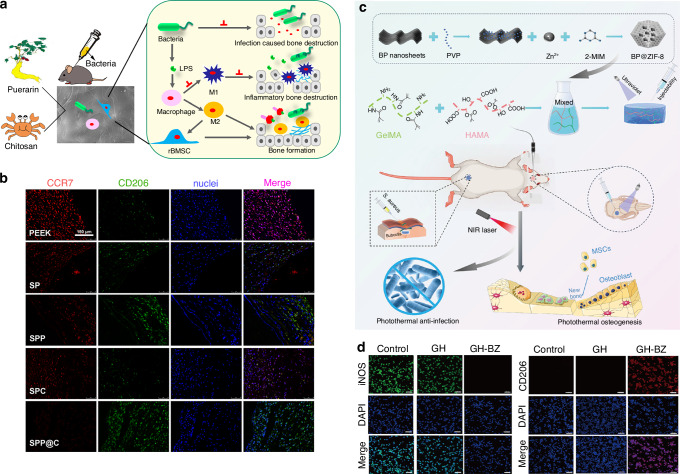
Nanocomposite materials with M2 macrophage polarization-regulating properties. **a** Schematic illustration of Puerarin@Chitosan composite for tissue repair after bacterial infection. **b** Dexamethasone-loaded nanocomposites promoting the expression of the M2 macrophage marker CD206 and suppressing the expression of the M1 macrophage marker CCR7 in vitro (scale = 150 μm). Reproduced with permission from ref. ^317^ Copyright 2022, KeAi Publishing Communications Ltd. **c** Preparation and functional workflow of injectable hydrogel (GelMA/HAMA/BP@ZIF-8). **d** GelMA/HAMA/BP@ZIF-8 significantly suppressing the expression of the M1 macrophage marker iNOS while promoting the expression of the M2 macrophage marker CD206 in vitro (scale = 50 μm). Reproduced with permission from ref. ^319^ Copyright 2024, American Chemical Society

Single nanomaterials have limited effects on regulating macrophage phenotypes. However, nanocomposites, which are constructed by combining various nanoscale materials with different physical and chemical properties, hold the promise of multifunctionality and precise regulation of macrophage polarization.^311^ In recent years, the impact of nanocomposites on macrophage polarization has been shown to have potential therapeutic value for a variety of diseases, including rheumatoid arthritis,^312^ osteoarthritis,^313^ osteoporosiss,^314^ wound infections,^315^ and cancer.^316^ In the field of bone infection research, Liu and colleagues^317^ developed a nanowire composite based on chitosan and puerarin, which exhibited excellent capabilities in promoting M2 anti-inflammatory polarization and bone regeneration in the rat model of infectious bone defects (Fig. 9a, b). Moses et al.^318^ loaded dexamethasone onto the surface of porous nanocomposites to achieve sustained and slow release, inducing M2 macrophage polarization and MSC adhesion, making it an effective strategy for treating implant-related infections. Additionally, nanocomposites based on ZIF-8 and black phosphorus nanosheets (BPNs) loaded into GelMA/HAMA hydrogels can induce M2 macrophage polarization and promote osteogenic differentiation in vitro. Furthermore, studies in a rat calvarial defect model confirmed the multifunctionality of hydrogel in antibacterial activity, inflammation suppression, and bone regeneration.^319^ (Fig. 9c, d). Compared to traditional single drugs and nanomaterials, nanocomposites have demonstrated potential for the treatment of a wide range of skeletal bacterial infections.

### Chronic infections: inducing pro-inflammatory macrophage phenotypes to promote pathogen eradication

Unlike the microenvironment of acute infections, pathogenic bacteria in chronic bone infections employ various strategies to evade recognition and destruction by immune cells, such as adhesion and colonization, biofilm formation, intracellular infections (involving osteoblasts, osteoclasts, bone cells, and immune cells), persistence cells, and SCVs, further exacerbating the severity of the infection.^320^ In addition to causing antibiotic treatment failures, biofilms and intracellular infections drive macrophages toward the M2 phenotype, weakening their bactericidal capacity while promoting abscess formation and increasing the complexity of treatment.^100^ Therefore, during the chronic stage of bone infections, it is crucial to use biomaterials that induce M1 polarization of macrophages, facilitating the secretion of pro-inflammatory factors and immune activation, reducing abscess formation, and promoting the eradication of biofilms and intracellular bacteria (Table 4).

**Table 4:** Summary of advanced biomaterials for modulating macrophage pro-inflammatory polarization in chronic bone infections

Biomaterials	Major components	In vitro model	In vivo model	Macrophage polarization	Functions	Ref.
Cu-incorporated SPEEK	SPEEK, Cu NPs	RAW264.7 cells and rBMSCs	Mice and rats implant-associated infection models	M1 polarization	In vitro: (1) Excellent direct bactericidal effects; (2) The biomaterial activates M1 macrophage polarization, enhancing phagocytic activity against MRSA. In vivo: Multimodal effects, including direct antibacterial activity and enhanced bone repair.	^325^
3D-printed Ti6Al4V implants modified by copper, and strontium ions	Ti6Al4V, copper, strontium	RAW264.7 cells and MC3T3-E1 cells	/	M1 polarization	In vitro: (1) The release of Cu²⁺ promotes M1 macrophage polarization, inducing pro-inflammatory responses to suppress infection; (2) Sr²⁺ promotes macrophage secretion of osteogenic factors, inducing osteogenic responses.	^326^

*Cu NPs* Copper nanoparticles, *MRSA* Methicillin-resistant *staphylococcus aureus*, *rBMSCs* Rat bone marrow-derived mesenchymal stem cells, *SPEEK* Sulfonated polyether ether ketone

**Fig. 10:**
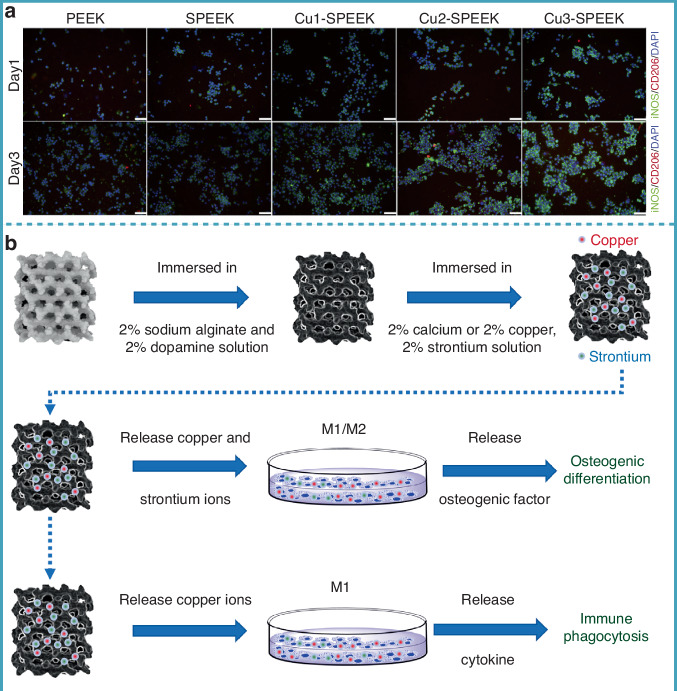
Biomaterials for modulating pro-inflammatory M1 macrophage polarization in chronic bone infections. **a** Cu-incorporated SPEEK promoting M1 macrophage polarization in vitro (scale = 50 μm). Reproduced with permission from ref. ^325^ Copyright 2019, Elsevier. **b** Schematic illustration of the preparation and macrophage polarization modulation of Cu-Sr modified Ti-6Al-4V implants. Reproduced with permission from ref. ^326^ Copyright 2023, Frontiers

In addition to their excellent bactericidal ability against drug-resistant strains, metal ions with immunomodulatory activity can also induce immune activation to enhance antibacterial efficacy, offering unique advantages in the treatment of chronic infections.^321^ For instance, copper, an essential trace element in the human body, plays a vital role in bone metabolism and innate immunity.^322^ Studies have reported that copper deficiency impairs the bactericidal function of macrophages in mice, increasing their susceptibility to infectious diseases.^323,324^ Liu et al.^325^ demonstrated that by immobilizing Cu NPs on the surface of SPEEK, a dual bactericidal effect against MRSA was achieved through contact and trapping mechanisms, while simultaneously inducing M1 macrophage polarization and enhancing their phagocytic capacity in vitro (Fig. 10a, b). In our research, Cu^2+^ were modified onto the surface of 3D-printed Ti-6Al-4V implants, where the rapid release of Cu^2+^ promoted pro-inflammatory M1 macrophage polarization, enhancing the ability of macrophages to phagocytize *S. aureus*, while also inducing immune-regulated osteogenic differentiation^326^ (Fig. 10c). Additionally, iron and zinc ions have been shown to promote M1 macrophage polarization,^327,328^ which is crucial for reversing immunosuppressive states and enhancing bactericidal activity in chronic bone infections.

### Sequential regulation of transition from M1 pro-inflammatory to M2 anti-inflammatory state

Although prolonged or excessive inflammatory cytokines can lead to chronic inflammation and impair tissue healing and repair, TNF-α and IL-6 are crucial for early tissue healing in mouse models of skin and skeletal muscle injury.^329^ In skeletal infections, an appropriate early pro-inflammatory response mediated by M1 macrophages is beneficial for immune activation and infection control.^325^ Therefore, researchers have recently begun to explore the sequential regulation of macrophage phenotypes by biomaterials to achieve orderly infection control and tissue repair. Specifically, biomaterials are designed to induce early M1 macrophage polarization, which activates the immune response to fight infection. Following infection control, macrophages are then regulated to transition from the M1 pro-inflammatory state to the M2 anti-inflammatory state, promoting the secretion of anti-inflammatory cytokines and facilitating bone tissue repairing.

**Fig. 11:**
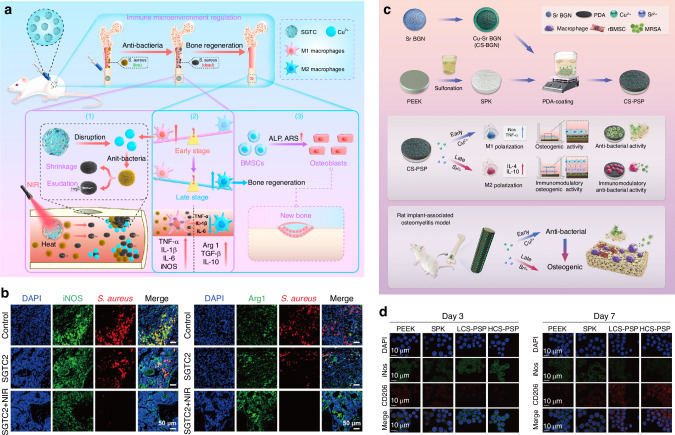
Biomaterials with sequential regulation of macrophage polarization from the pro-inflammatory M1 state to the anti-inflammatory M2 state. **a** Schematic illustration of the mechanism of multifunctional SGTC2 microspheres sequentially regulating macrophage polarization for osteomyelitis treatment. **b** SGTC2 microspheres suppressing the expression of the M1 macrophage marker iNOS at 3 weeks and promoting the expression of the M2 macrophage marker Arg-1 at 6 weeks during osteomyelitis treatment. Reproduced with permission from ref. ^330^ Copyright 2024, American Chemical Society. **c** Schematic illustration of CS-BGNs-PDA-coated sulfonated PEEK (CS-PSP) sequentially regulating macrophage polarization in vitro and in vivo. **d** Immunofluorescence staining showing that CS-PSP promotes M1 macrophage polarization at 3 days and M2 macrophage polarization at 7 days. Reproduced with permission from ref. ^331^ Copyright 2023, Wiley-VCH

For persistent, deep, and complex infections in bone tissue caused by *S. aureus*-induced osteomyelitis, a synergistic therapy that combines immunomodulation with physical and chemical methods is a promising strategy. For example, a hydrogel composed of sodium alginate (SA) ionically cross-linked with genipin (Gp)-cross-linked gelatin (Gel), loaded with tannic acid (TA) and Cu^2+^, exhibits strong antibacterial and anti-biofilm properties under 808 nm near-infrared (NIR) stimulation. On the other hand, it sequentially regulates macrophage phenotypes by releasing TA and Cu^2+^. In the early stage of osteomyelitis, it stimulates M1 macrophage polarization, which secretes pro-inflammatory cytokines (TNF-α, IL-1β, and IL-6) to combat infection. Then, in the mid-to-late stage, the macrophages transition to the M2 phenotype, which promotes tissue repair and stimulates osteogenic differentiation of BMSCs (Fig. 11a). In a mouse femoral osteomyelitis model, the hydrogel induced the recruitment of M1 macrophages to the infection site during the early implantation stage (3 weeks) to eliminate the infection, and enhanced M2 macrophage polarization during the mid-to-late stage (6 weeks), thereby establishing a favorable dynamic immune microenvironment for bone tissue regeneration^330^ (Fig. 11b). Similarly, the combined release of metal ions can also achieve macrophage phenotype switching. For example, copper-strontium bioactive glass nanoparticles (Cu-Sr BGNs) modified on the surface of PEEK enable the sequential release of Cu^2+^ and Sr^2+^, leading to macrophage phenotype switching. Specifically, the early release of Cu^2+^ from the outer layer induces M1 macrophage polarization, while the later release of Sr^2+^ from the inner layer induces M2 macrophage polarization, facilitating a transition from antibacterial activity to bone tissue repair^331^ (Fig. 11c, d). Although in vivo evidence for the sequential regulation of macrophage polarization by the material is lacking, this combined immunomodulatory strategy exhibited excellent bacterial clearance and osseointegration in a rat calvarial defect model. Furthermore, regulating macrophage phenotype switching with a single drug is challenging. Combining drugs with different regulatory functions offers a viable strategy for sequential modulation of macrophages. For instance, Ma et al.^332^ developed a hydrogel containing W9 peptide and LL-37 peptide-loaded microspheres for filling irregular bone defects. The early release of LL-37 promoted M1 macrophage pro-inflammatory activity, aiding in infection prevention. Subsequently, the release of the W9 peptide downregulated pro-inflammatory cytokine expression, inducing the transition of macrophages to the M2 phenotype, which in turn promoted osteogenic differentiation of BMSCs in vitro. However, implantation in the rat bone defect model for 1 month significantly promoted M2 macrophage polarization, while at 3 months, iNOS expression was mildly upregulated. This differs from the in vitro immunoregulatory results and may be due to the limited immunoregulatory function of the material on macrophages in the bone defect environment without bacterial infection.

In the future, these strategies may become a key research direction for developing biomaterials that combat bone infections. However, the sequential regulation strategy of macrophage polarization in the bone infection microenvironment still faces numerous challenges. Firstly, the timing window for polarization must be precisely controlled.^333^ Specifically, biomaterial-mediated macrophage polarization must be tightly aligned with the progression stage of bone infection; otherwise, inadequate immune clearance may lead to infection recurrence. Developing accurate biomarkers for dynamic monitoring based on different stages of bone infection, along with designing immunomodulatory biomaterials capable of dynamic degradation and release, may represent an important future research direction. Secondly, the heterogeneity of the bone infection microenvironment may interfere with the functionality of the materials. Lesions in bone infection are often accompanied by hypoxia, acidic pH, and elevated ROS levels, which can affect material degradation and the release of drugs or bioactive factors. To address this, designing infection-responsive materials (such as pH- or ROS-sensitive hydrogel) can enable environment-triggered, on-demand release of polarization factors. Furthermore, the heterogeneity among preclinical animal models (e.g., rat femoral osteomyelitis and implant-associated infection models) makes it difficult to fully replicate the human bone infection microenvironment. For instance, in diabetic patients, hyperglycemia promotes macrophage polarization toward the M1 phenotype, exacerbating tissue damage.^334^ Moving forward, technological complementarity (such as organoid screening combined with primate validation) and interdisciplinary integration (such as AI-based dynamic monitoring and gene editing)^335^ will be crucial to accelerating the clinical translation of macrophage polarization-regulating biomaterials for the treatment of bone infections.

### Sequential regulation of transition from M1 pro-angiogenesis to M2 pro-vascularization and -osteogenesis states

After the implantation of biomaterials, the osseointegration effect at bone infection sites is regulated by macrophage-related cytokines, which sequentially initiate vascularization and bone formation.^336^ The timely appearance of blood vessels is a critical step in bone regeneration, as it provides nutrients, oxygen, and essential ions for the differentiation of stem cells into OBs.^337^ A previous study has reported that M1 macrophages secrete VEGF to initiate the process of angiogenesis, while M2 macrophages are responsible for secreting vascular growth-related factors such as PDGF-BB.^338^ In addition to activating immune responses to combat infection, M1 macrophage polarization also contributes to angiogenesis. However, prolonged inflammation can impair and delay angiogenesis and subsequent osteogenesis.^339^ Thus, biomaterials that can timely regulate macrophage subtypes are crucial for the formation and growth of blood vessels, significantly influencing bone tissue healing. Inspired by the regulatory effects of Ca²⁺, Mg²⁺, Sr²⁺, and Zn²⁺ on the M2 macrophage phenotype in bone minerals, Yu et al.^340^ used a hydrothermal method to prepare a sodium titanate coating co-doped with four ions. The results demonstrated that the quadruple ions initially induced M1 macrophage polarization, promoting angiogenesis by acting on endothelial cells (ECs). Subsequently, they induced the transition of macrophages to the M2 phenotype, achieving rapid vascularization and osteogenic differentiation in vitro. Mechanistically, the TLR4-NF-κB signaling pathway was involved in the regulation of M1 macrophage polarization by the ions, while the CaSR-PI3K-Akt1 signaling pathway was enhanced during the M2 macrophage polarization process. In a rat implantation-related model, the material induced M2 macrophage polarization and enhanced CD31 expression during the early post-implantation period (days 1 and 3), while promoting the expression of osteogenesis-related proteins COL-2 and OPN by day 7.

A dual-drug release system plays a crucial role in regulating the polarization of macrophages from the M1 to M2 phenotype, enabling sequential modulation through controlled release timing. Spiller and colleagues^341^ designed a decellularized bone scaffold loaded with interferon-gamma (IFN-γ) and IL-4, successfully achieving sequential regulation of vascularization and osteogenesis in vitro. Specifically, the early release of IFN-γ enhanced M1 macrophage polarization, promoting the expression of VEGF, which led to vascular infiltration. The slow release of IL-4 subsequently facilitated the transition to M2 macrophage polarization, which in turn secreted PDGF-BB, recruiting pericytes to further promote vascularization. In the in vivo model, 2 weeks after implantation of the bone scaffold, the endothelial cell marker CD31 was significantly upregulated, whereas no notable changes were observed in macrophage M1/M2 polarization. Qiao et al.^342^ further established a co-culture system consisting of primary BMMs, MSCs, and ECs to investigate the immunomodulatory synergy of biomaterials in regulating bone healing post-implantation. The results demonstrated that early M1 macrophage polarization promoted the recruitment of osteogenic and angiogenic progenitor cells, while later M2 macrophage polarization facilitated bone mineralization and vascular maturation. This sequential activation pattern promoted a highly coordinated bone healing process (Table 5). However, these results also suggest that in the complex in vivo bone tissue microenvironment, the induction of M1 macrophage polarization and the promotion of angiogenesis do not fully correspond and may undergo dynamic changes. Therefore, how to orchestrate the sequential regulation of these metal ions to exert precise modulatory functions in response to microenvironmental changes remains a key design challenge for macrophage polarization-regulating biomaterials.

**Table 5:** Summary of advanced biomaterials for sequential regulation of transition from M1 to M2 state

				Early stage		Mid-to-late stage		
Biomaterials	Major components	In vitro model	In vivo model	Polarization phenotype	Functions	Polarization phenotype	Functions	Ref.
Multifunctional injectable microspheres	SA and Gp-cross-linked Gel incorporated with TA and copper ions (Cu^2+^)	RAW264.7 cells and BMSCs	Rat osteomyelitis model	M1 polarization	In vitro: (1) The release of Cu^2+^ promotes M1 macrophage polarization and upregulates the expression of pro-inflammatory factors; (2) The release of Cu^2+^ can also induce bacterial DNA damage, achieving an antibacterial effect. In vivo: It induces M1 macrophage polarization to clear *Staphylococcus aureus* at 3 weeks.	M2 polarization	In vitro: (1) The release of TA promotes M2 macrophage polarization, upregulates the expression of anti-inflammatory factors, and facilitates the osteogenic differentiation of BMSCs; (2) The release of TA also exhibits significant antibacterial and antioxidant functions. In vivo: It induces M2 macrophage polarization to promote bone tissue repair and regeneration at 6 weeks.	^330^
Functionalized PEEK	PEEK, CS-BGNs, PDA	RAW264.7 cells and rBMSCs	Mice implant-related osteomyelitis model	M1 polarization	In vitro: (1) The rapid release of outer-layer Cu^2+^ induces M1 macrophage polarization, facilitating early antibacterial activity; (2) It promotes early osteogenic differentiation.	M2 polarization	In vitro: (1) The slow release of inner-layer Sr^2+^ promotes M2 macrophage polarization, alleviating inflammatory responses; (2) It enhances mid-to-late-stage osteogenic differentiation and bone mineralization.	^331^
Microsphere−Gel composite system	Porcine SIS hydrogel, LL37 peptides, PLGA microspheres, WP9QY (W9) peptide	RAW264.7 cells and BMSCs	Rat model of bone defect	M1 polarization	In vitro: (1) The release of LL37 peptides promotes M1 macrophage polarization, providing early infection prevention; (2) The release of LL37 peptides also increases the number of BMSCs. In vivo: It induces M2 macrophage polarization to promote bone tissue repair and regeneration at 1 month.	M2 polarization	In vitro: (1) The release of W9 peptides promotes M2 macrophage polarization, alleviating inflammatory responses; (2) The release of W9 peptides also enhances the osteogenic differentiation of BMSCs. In vivo: It mildly induces M1 macrophage polarization but does not affect the final osteogenic outcome.	^332^
Cubic multi-ions-doped Na_2_TiO_3_ nanorod-like coatings	Ca^2+^, Mg^2+^, Sr^2+^, Zn^2+^, Na_2_TiO_3_ nanorod-like coatings	RAW264.7 cells, HUVECs, and rBMSCs	Rat implant-related model	M1 polarization	In vitro: Ion release induces M1 macrophage polarization, promoting angiogenesis by acting on endothelial cells. In vivo: It induces M1 macrophage polarization and enhances angiogenesis.	M2 polarization	In vitro: Ion release induces M2 macrophage polarization, promoting osteogenic differentiation. In vivo: It induces M2 macrophage polarization and facilitates vascularized osteogenesis.	^340^
Scaffolds based on decellularized bone	IFN-γ, IL4, decellularized bone scaffolds	Monocyte-derived macrophages	Mice model of subcutaneous implantation	M1 polarization	In vitro: The short-term release of IFN-γ induces M1 macrophage polarization, promoting VEGF expression and vascular infiltration. In vivo: It enhances angiogenesis, while no differences are observed in macrophage phenotype.	M2 polarization	In vitro: The sustained release of IL-4 induces M2 macrophage polarization, further promoting the secretion of PDGF-BB and angiogenesis.	^341^

*BMSCs* Bone marrow-derived mesenchymal stem cells, *CS-BGNs*: Cu-Sr bilayer bioactive glass nanoparticles, *Gel* Gelatin, *Gp* Genipin, *HUVECs* Human umbilical vein endothelial cells, *IFN-γ* Interferon gamma, *IL-4* Interleukin-4, *PDA* Polydopamine, *PDGF-BB* Platelet-derived growth factor-BB, *PEEK* Polyether ether ketone, *PLGA* Poly(lactic-co-glycolic acid), *ROS* Reactive oxygen species, *SA* Sodium alginate, *SIS* Small intestinal submucosa, *TA* Tannic acid, *VEGF* Vascular endothelial growth factor

## Challenges and prospects

**Fig. 12:**
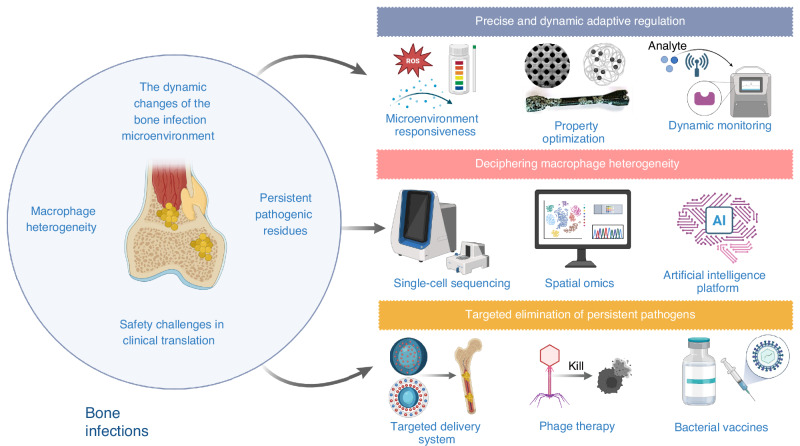
Current challenges and corresponding strategies in macrophage polarization-regulating biomaterials. Future macrophage polarization-regulating biomaterials can focus on precise and dynamic adaptive regulation, deciphering macrophage heterogeneity, and targeted elimination of persistent pathogens, providing support for the development of novel therapies for bone infections and a deeper understanding of disease mechanisms. Created with BioRender.com

Focusing on the dynamic changes in macrophage phenotypes within the infectious microenvironment and achieving precise regulation of macrophage polarization by continuously optimizing the physicochemical properties of biomaterials is a promising strategy for treating bone infections. This review first provides a detailed description of the immune evasion strategies of bacteria in the bone infection microenvironment and the role of macrophage polarization. It then discusses how various physicochemical properties of biomaterials influence the fate of macrophages. Additionally, we summarize targeted strategies, research advancements, and challenges in regulating macrophage polarization at different stages of bone infection following biomaterial implantation, while also discussing the future direction of biomaterial-mediated macrophage immunotherapy. However, over the past decade, there have been many advanced attempts and applications of biomaterial-mediated macrophage polarization remodeling and sequential regulation strategies in the treatment of bone infection, promising significant progress and development. Nevertheless, some challenges remain to be addressed for successful clinical translation (Fig. 12):

1. The imbalance between anti-inflammatory and pro-inflammatory phenotypes, leading to refractory abscesses and cytokine storms, poses significant challenges in regulating macrophage polarization. Biomaterials aimed at treating bone infections by modulating macrophage phenotypes must maintain two balances: (i) In acute bone infections, a balance between pathogen elimination mediated by pro-inflammatory phenotypes and the risk of excessive inflammatory responses. While pro-inflammatory cytokines released during the acute phase facilitate rapid bacterial clearance, prolonged and excessive inflammatory responses may lead to bacterial mutations, triggering cytokine storms. Ideally, biomaterials at this stage should possess the ability to monitor cytokine levels, enabling adaptive and dynamic regulation of pro-inflammatory cytokine secretion to avoid excessive inflammation and tissue damage; (ii) In chronic bone infections, a balance between bone regeneration mediated by anti-inflammatory phenotypes and the risk of excessive fibrosis and recurrence of residual bacteria. Specifically, M2 macrophage phenotypes help mitigate inflammatory responses and accelerate tissue repair, but high levels of anti-inflammatory cytokines during chronic infection hinder rapid bacterial clearance, potentially resulting in persistent bacteria in bone cells and tissues, which is linked to the recurrence of osteomyelitis. An effective approach is to optimize the characteristics of biomaterials continuously, ensuring complete bacterial clearance before promoting M2 macrophage polarization. Therefore, it is essential to consider and design biomaterials that can achieve self-dynamic and precise regulation based on changes in the microenvironment (cytokine levels) in the future.
2. Currently, descriptions of M1 and M2 macrophage polarization remain limited to initial in vitro observations. Recent detailed macrophage phenotyping has altered the understanding of the traditional M1/M2 dichotomy.^57,343^ Given the complexity and dynamics of the infectious immune microenvironment, the rapid advancements in high-throughput sequencing technology indicate that future research should focus on the precise detection of macrophage states and functions at different stages of infection in vivo. Recently, researchers have identified two new subpopulations of neutrophils, CD10^-^CD64^+^PD-L1^+^ and CD10^-^CD64^+^CD16^low/-^CD123^+^, which show specificity in distinguishing between sepsis and sterile inflammation.^344^ Utilizing single-cell and spatial omics technologies to explore the heterogeneity and functional subgroups of macrophages can guide more refined macrophage immunotherapy for the clearance of resistant bacteria. Additionally, detailed mechanistic exploration using omics technologies is essential for comprehensively understanding the immune microenvironment changes at different stages of infection. Therefore, employing more complex and dynamic models to describe the functional states of macrophages within the infectious microenvironment will aid in the design and development of novel macrophage-regulating biomaterials.
3. Traditionally, the eradication of bacterial infections in bones has primarily focused on the direct removal of bacteria and biofilms, often overlooking bacteria that exist in the forms of “persistent cells” and SCVs. Recent clinical cases and animal models have revealed that bacterial invasion of the OLCN is a key factor in the recurrence of osteomyelitis.^345,346^ However, targeted treatments for intracellular infections, SCVs, and OLCN invasion in bone infections pose significant challenges, particularly due to the potential induction of bacterial resistant phenotypes by traditional antibiotics and the lack of in vivo models for studying bacterial invasion of the OLCN. Recent studies have investigated the molecular mechanisms of *S. aureus* invasion of OLCN through an in vitro model named SiM-CA, identifying potential molecular targets such as pbp3 and pbp4.^347,348^ Therefore, it is essential to establish reliable in vivo OLCN models to explore specific molecular targets for bacterial invasion using high-throughput sequencing and molecular biology techniques. Moreover, emphasizing deeper targeted bactericidal strategies for intracellular bacteria and OLCN invasion is crucial. Currently, bone-targeting strategies such as bisphosphonate-conjugated antibiotics and phage therapy are being studied. Mouse models of osteomyelitis have demonstrated successful targeting and release of bisphosphonate-bound antibiotics, which remain stable in serum.^349^ Phage therapy has been initially applied and successfully used in wound infections and diabetic foot ulcers^350,351^; however, it is limited by small sample sizes. Future efforts should focus on integrating these with biomaterials to enhance their specificity for bone and cells, allowing for the precise clearance of bacteria residing within cells and bone tissues.
4. The physicochemical properties (such as topography, wettability, charge, chirality, and composition) of biomaterials can actively regulate macrophage fate, inducing their participation in antibacterial and tissue repair processes. However, due to significant differences in in vitro models, the specific mechanisms of interaction between physicochemical properties and macrophages remain unclear, with some contradictory viewpoints emerging. Therefore, it is essential to study the detailed molecular mechanisms by which the physicochemical properties of biomaterials regulate macrophage fate, using in vivo infectious microenvironments and quantitative methods. Additionally, there is limited research in specific areas such as macrophage biomechanics and spatial constraints.^352,353^ Future investigations into the relationship between these signals and macrophage polarization and activation will enhance our understanding of the response mechanisms of macrophages in complex infectious microenvironments and guide the design of immunomodulatory biomaterials.
5. Safety is a crucial aspect that must be comprehensively assessed before the clinical translation of biomaterials. Currently, the side effects of biomaterials on immune cells are not well understood. For instance, recent studies have primarily focused on how biomaterials induce cuproptosis in bacteria through the release of Cu ions,^354,355^ neglecting the potential impact of elevated copper ion concentrations on immune cell function. On the other hand, although bacterial vaccines have shown promising results in activating and enhancing innate and adaptive immune responses, their clinical translation is limited by regulatory approvals.^356^ Therefore, it is essential to seek vaccine components that exhibit high immunogenicity, specificity, and safety. Additionally, with advancements in 3D/4D printing technology, biomaterial-assisted vaccines can be personalized and customized to modulate macrophage behavior, contributing to safe and precise treatments for bone infections.

## Supplementary information

Permission of copyright
